# Pregnancy alters fatty acid metabolism, glucose regulation, and detoxification of the liver in synchrony with biomechanical property changes

**DOI:** 10.1016/j.heliyon.2024.e39674

**Published:** 2024-10-22

**Authors:** Jing Guo, Karolina Krehl, Yasmine Safraou, Iwona Wallach, Jürgen Braun, David Meierhofer, Ingolf Sack, Nikolaus Berndt

**Affiliations:** a Department of Radiology, Charité – Universitätsmedizin Berlin, corporate member of Freie Universität Berlin and Humboldt-Universität zu Berlin, Berlin, Germany; b Department of Veterinary Medicine, Institute of Animal Welfare, Animal Behavior and Laboratory Animal Science, Freie Universität Berlin, Berlin, Germany; c Institute of Computer-assisted Cardiovascular Medicine, Deutsches Herzzentrum der Charité, Berlin, Germany; d Charité – Universitätsmedizin Berlin, corporate member of Freie Universität Berlin and Humboldt-Universität zu Berlin, Berlin, Germany; e Institute of Medical Informatics, Charité – Universitätsmedizin Berlin, corporate member of Freie Universität Berlin and Humboldt-Universität zu Berlin, Berlin, Germany; f Mass Spectrometry Facility, Max Planck Institute for Molecular Genetics, Berlin, Germany; g Department of Molecular Toxicology, German Institute of Human Nutrition Potsdam-Rehbruecke (DIfE), Nuthetal, Germany

**Keywords:** Proteomics analysis, Kinetic models, Metabolic liver reprogramming, Magnetic resonance elastography, Tissue biomechanics

## Abstract

Pregnancy places a metabolic burden on the body including the liver, which is responsible for ensuring adequate nutrition for the maternal and fetal systems. To gain a better understanding of liver adaptation, this study investigates metabolic shifts occurring in livers of pregnant rats. Metabolic capacities of the livers of pregnant and non-pregnant female Wistar rats were assessed using comprehensive metabolic models. Kinetic metabolic models were generated for each animal based on protein abundance data from proteomics analysis allowing for a subject-specific assessment of hepatic metabolic functions. Data are available via ProteomeXchange with identifier PXD050758. Additionally, tissue stiffness, viscosity, and water diffusion obtained from magnetic resonance imaging and elastography were correlated with metabolic capabilities to study the relationship between metabolic function and biophysical properties. Proteome profiling revealed differences in protein expression in the livers of pregnant and non-pregnant animals. Functional analysis showed significant variations in metabolic capacities. Livers of pregnant rats had reduced capacities in carbohydrate and fatty acid metabolism, along with altered urea synthesis. Additionally, there were associations between metabolic functions and biophysical properties highlighting potential links between changes in liver structure and metabolic capacities during pregnancy. In summary, our work reveals extensive hepatic metabolic changes in pregnant rats. The liver adapts its metabolic capacities to ensure whole-body metabolic homeostasis but may struggle to counteract nutritional challenges, such as hypoglycemia. The study, employing a personalized approach combining proteomics, kinetic modeling, and advanced imaging, sheds light on the intricate interplay between hepatic adaptations and medical imaging markers, providing a foundation for further investigations into the implications for maternal and fetal health.

## 1 Introduction

During pregnancy, metabolism must change drastically to ensure an adequate supply of amino acids, nucleotides, fatty acids, and sugars, which are necessary to support the growth and development of the fetus, in the maternal and fetal body. Multiple alterations in whole-body metabolism have been observed during pregnancy including the availability and utilization of nutrients like glucose, fatty acids, or ketone bodies, as well as changes in the circulating hormones like insulin, glucagon, and estrogen, all contributing to systemic metabolic adaptations [[1], [2], [3]].

The liver is one of the central metabolic organs responsible for the maintenance of plasma nutrients such as glucose. It enables growth by providing important building blocks to the rest of the body, e.g. in the form of lipoproteins, and the detoxification of potentially harmful substances like ammonia. An important feature of hepatic metabolism is the ability to adapt its functional state according to altered demand, changes in circulating plasma nutrients, and variable hormonal signaling by insulin and glucagon, all three representing important mechanisms for the regulation of hepatic metabolism [[4], [5], [6]]. Therefore, it is plausible to assume that during pregnancy, changes in liver metabolism should contribute significantly to metabolic alterations observed in whole-body metabolism. However, the contributions of the liver to overall metabolic changes during pregnancy are poorly understood.

While increased hepatic gluconeogenesis has been reported for lactating animals [7], the reports during pregnancy are less clear. Various studies report a moderate increase in basal endogenous hepatic glucose production (see Butte, 2000 [1], and references within), but there are also indications of reduced gluconeogenesis during pregnancy [8]. Smith [9] reported that the ratio of hexokinase and glucokinase to glucose 6-phosphatase as well as the ratio of pyruvate kinase to phosphoenolpyruvate carboxykinase changed in favor of the glycolytic enzymes indicating increased glycolytic capacity. This is in line with the findings by Rathgen et al. [8] where increased activity of pyruvate kinase and hexokinase and decreased activity of glucose 6-phosphatase were reported. Analysis of intrahepatic metabolite concentrations showed a significant decrease in the amounts of glucose 6-phosphate, fructose 6-phosphate, dihydroxyacetone phosphate, and fructose 1,6-bisphosphate in the livers of pregnant rats, but this might be a consequence of the overall reduced glucose content in the liver and the reduced glucose level in the peripheral blood [8]. Also, Brockerhoff et al. [10] did not find significant alterations in hepatic glycogen content or the activities of the significant enzymes of glycogen metabolism during pregnancy.

Besides changes in hepatic glucose metabolism, pregnancy leads to alterations in circulating concentrations of triacylglycerol (TAG), fatty acids, cholesterol, and phospholipids [11]. Initially, during the first eight weeks, these lipids and lipoproteins decrease, but they gradually rise throughout the second and third trimesters. Total and LDL cholesterol decrease early in pregnancy before increasing later, while HDL cholesterol rises by week 12 and remains elevated. VLDL and TAG follow a similar pattern, decreasing in the first eight weeks and then increasing until term [1]. The increase in plasma estrogen during late gestation has been shown to enhance liver production of VLDL [12] and decrease the expression and activity of lipoprotein lipase (LPL) in the liver [13], redirecting circulating lipoproteins to the placenta where LPL activity is increased.

Investigations about other metabolic pathways in the liver during gestation are scarce. Alterations in the citric acid cycle were examined by Brockerhoff et al. [14] but no correlation with gestational age could be found, so alterations in hepatic central energy metabolism remain elusive. The metabolic changes occurring in other pathways of hepatic carbohydrate metabolism as well as the detoxification of ammonia and ethanol have not been investigated.

In this work, we used proteomic data sets obtained from the livers of non-pregnant and pregnant rats for kinetic modeling of central liver metabolism. Using HEPATOKIN1 [15], a kinetic model of central hepatic metabolism, we assess the changes occurring in hepatic central metabolism including carbohydrate metabolism, lipid metabolism, amino acid metabolism as well as ammonia and alcohol detoxification. Assessing the metabolic capacities of the livers, we show that during late pregnancy, there are significant changes in the metabolic capacities of carbohydrate and fatty acid utilization pathways, as well as for urea synthesis and ethanol detoxification.

Furthermore, the maternal liver, as a central metabolic organ, undergoes significant structural and hemodynamic changes during pregnancy, which are reflected by changes in biomechanical properties [16,17]. It has also been reported that liver stiffness independently predicts preeclampsia [16,18]. In previous work, we found that biophysical, tissue-structure-related imaging markers are sensitive to changes in liver tissue during pregnancy [19]. As our recent findings indicate a correlation between hepatic metabolic function and biophysical tissue properties quantified by magnetic resonance elastography (MRE) and diffusion-weighted imaging (DWI) [20,21], we aimed through *ex vivo* tissue analysis using an animal model (i) to demonstrate the metabolic adaptations that the liver undergoes during pregnancy, and (ii) to show that medical imaging can detect changes in the biophysical properties of the liver associated with metabolic changes.

## 2 Results

### 2.1 Proteomics

Proteome profiling yielded protein intensities for 5004 proteins that were identified in the livers of pregnant and control rats. Statistical analysis revealed 738 upregulated and 330 downregulated proteins, corresponding to 21.3%of the detected proteome to be differentially expressed in the livers of pregnant rats. To analyze the implications of protein abundance changes for hepatic metabolic functions, we used Hepatokin1. We used the protein abundance data of 354 metabolic enzymes and transporters obtained from LC-MS/MS analysis to generate individual instantiations of the metabolic model for each liver. Out of these, 108 proteins corresponding to 30.5 % were significantly different between the livers of the two groups. The relative fraction of differentially expressed metabolic proteins was about 1.5 times higher than in the total proteome. About 55 % of the metabolic proteins had a lower abundance in the livers of pregnant rats than in control livers.

**Fig. 1:**
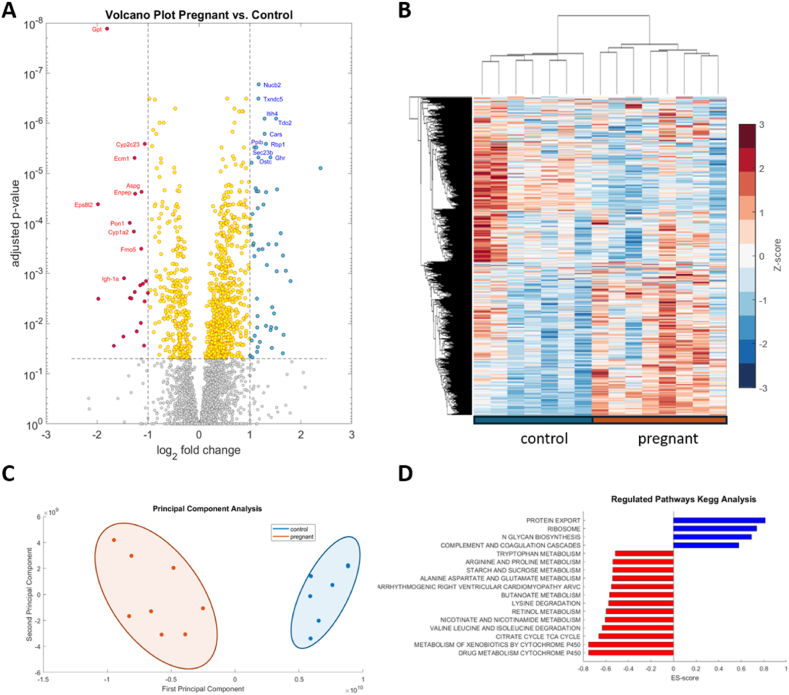
Biostatistics analysis of proteomic data. (A) Volcano plot showing log2 fold changes versus adjusted p-values assessed by two-sided *t*-test with Benjamini–Hochberg correction for multiple testing of all proteins detected in livers of pregnant animals (pregnant, n = 8) vs. livers of non-pregnant animals (control, n = 7). Negative log2 fold change (left side) corresponds to proteins that are downregulated in the livers of pregnant animals, while positive log2 fold changes (right side) correspond to upregulated proteins. Gray dots represent proteins that are not significantly different between the two groups (p-value >0.05), and yellow dots represent proteins that have a p-value <0.05 but a |log2 fold change| <1. Red dots represent proteins that are significantly downregulated (log2 fold change < -1, p-value <0.05), and blue dots represent proteins that are significantly upregulated (log2 fold change >1, p-value <0.05). The ten most significant up and downregulated proteins are indicated. (B) Hierarchical cluster analysis of all proteins and all samples normalized so that for each protein the mean is 0 and the standard deviation is 1. Red and blue colors indicate increased and decreased abundance of the respective protein in each sample compared to the overall mean. There is a perfect separation between the livers of control and pregnant animals. (C) Principal component analysis shows a clear separation between the livers of control and pregnant rats along the first two principal components. (D) Gene set enrichment analysis: Enrichment (ES) score of pathways upregulated (blue) and downregulated in livers of pregnant vs. control animals. (For interpretation of the references to color in this figure legend, the reader is referred to the Web version of this article.)

Fig. 1A shows the volcano plot of the whole proteome. Red (downregulated) and blue (upregulated) dots depict significantly differentially abundant proteins with p < 0.05 and |log2-fold change| >1. Among the top downregulated proteins (highlighted in red) are enzymes involved in blood pressure regulation, such as CYP2C23, a member of the CYP450 family that metabolizes arachidonic acid to epoxyeicosatrienoic acids, and glutamyl aminopeptidase (ENPEP), which converts angiotensin II to angiotensin III, influencing vasoconstriction. Additionally, enzymes crucial for drug and xenobiotic metabolism (CYP1A2, FMO5) and those involved in amino acid metabolism (GPT, ASPG) are also prominently downregulated. The upregulated proteins include NUCB2, which is involved in regulating food intake, energy expenditure, and glucose metabolism; immune response proteins, such as TDO2 and ITIH4; proteins related to protein synthesis regulation like CARS1, SEC23B, and OSTC; proteins involved in cellular matrix modulation, such as PPIB; and those essential for retinol metabolism, like RBP1. A complete list of differentially regulated proteins is given in the Supplementary Table S1. In line with these protein markers, gene set enrichment analysis (Fig. 1D) identified up and downregulated pathways in the livers of pregnant animals. While protein synthesis, ribosome pathway, and n-glycan biosynthesis are upregulated, multiple metabolic pathways for amino acids, as well as citric acid cycle and cytochrome p450-dependent metabolism of drugs and xenobiotics are downregulated. Unbiased cluster analysis of all proteins and samples (Fig. 1B) as well as principal component analysis (Fig. 1C) leads to a perfect separation between the livers of control and pregnant animals.

### 2.2 Carbohydrate metabolism in the livers of pregnant rats

**Fig. 2:**
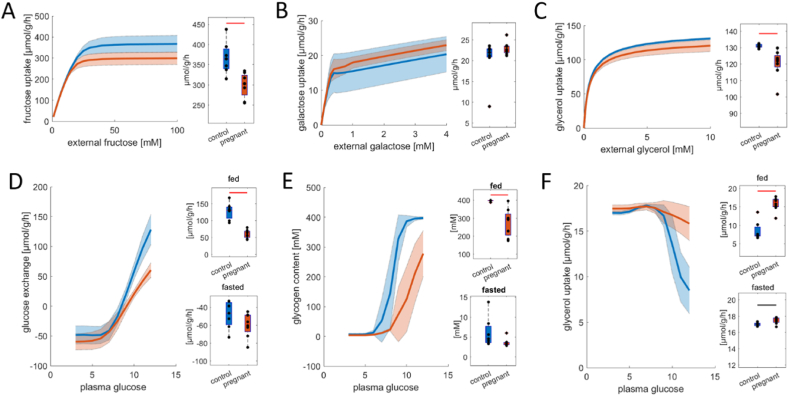
Metabolic adaptations of carbohydrate metabolism in livers of pregnant (n = 8) vs. non-pregnant (n = 7) rats. (A–C) Uptake rates of fructose (A), galactose (B), and glycerol (C) in dependence on substrate availability. Corresponding maximal capacities are depicted by the box plots on the right. The center lines represent the median, the boxes represent the interquartile range, and the whiskers are defined by values within 1.5 times the interquartile range. Outliers are represented as dots. Blue boxes show control livers, and red depict livers of pregnant animals. Black and red bars indicate significant differences assessed by a two-sided *t*-test with a p-value <0.05 and 0.01, respectively. (D–F) Metabolic functions under a wide range of physiological conditions: Glucose exchange (D), glycogen storage (E), and glycerol uptake (F) in dependence on plasma glucose availability. Blue curves depict control livers, while red curves depict the livers of pregnant animals. Solid lines depict mean values for each group, and shaded areas depict the standard variation. Box plots on the right depict metabolic functions under fed (12 mM plasma glucose) and fasted conditions (3 mM plasma glucose). Glucose exchange switches from gluconeogenesis (negative values) to glycolysis as plasma glucose levels increase. Glycolytic activity under fed conditions is decreased in the livers of pregnant animals. Glycogen storage increases with increased glucose availability but is much less pronounced in the livers of pregnant animals. Glycerol uptake is higher in pregnant rats, especially at high plasma glucose levels. (For interpretation of the references to color in this figure legend, the reader is referred to the Web version of this article.)

First, we assessed the maximal metabolic capacities of the livers for carbohydrate metabolism (see Materials and methods). Fig. 2A–C depict the metabolic capacities for the utilization of fructose (A), galactose (B), and glycerol (C). As can be seen, the capacity of livers of pregnant rats to metabolize fructose and glycerol is significantly decreased, while the capacity for galactose utilization is not significantly different from control animals (see box plots). Next, we assessed the metabolic functionality of the livers by systematically varying plasma glucose concentrations between 3 and 12 mM, spanning physiological conditions from fasted (low insulin, high glucagon, high fatty acids) to fed conditions (high insulin, low glucagon, low fatty acids) [22].

Fig. 2D–F depicts the glucose exchange flux, glycogen storage, and glycerol uptake in dependence on external glucose concentration. Livers of pregnant rats have a remarkably lower glycolytic activity at high plasma glucose concentrations, while their rate of gluconeogenesis at low plasma glucose concentrations is unchanged. The neutral point (i.e., the external glucose concentration at which the liver switches from glucose production to glucose consumption) is shifted from 8.3 mM to 9.1 mM for the livers of pregnant rats. Overall, this shows a shift toward gluconeogenesis and a decreased responsiveness of the liver during pregnancy to increased plasma glucose. This is also very clearly reflected in the hepatic glycogen storage depicted in Fig. 2C. While hepatic glycogen stores are replenished at ∼8 mM plasma glucose for the control liver, this is severely delayed in the livers of pregnant animals leading to an overall significantly decreased glycogen storage. In contrast, glycerol utilization is not decreased at high external glucose levels in contrast to control livers (Fig. 2F).

### 2.3 Fatty acid metabolism

**Fig. 3:**
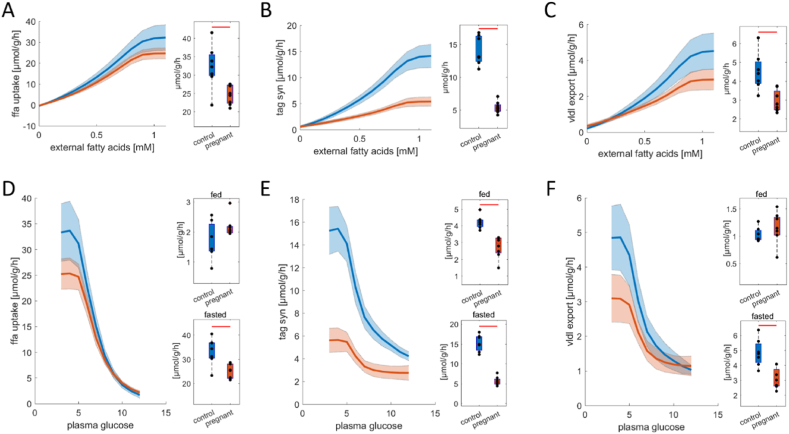
Metabolic adaptations of capacities for fatty acid metabolism in the livers of pregnant (n = 8) vs. non-pregnant (n = 7) rats. Capacities for (A) free fatty acid (ffa) uptake, (B) triacylglycerol (tag) synthesis, and (C) lipoprotein synthesis (vldl export) in dependence on plasma fatty acid concentrations. Corresponding maximal capacities are depicted by the box plots on the right. Metabolic functions for fatty acid metabolism in pregnant animals under physiological conditions from fasted (3 mM plasma glucose) to fed (12 mM plasma glucose) states. (D) Ffa uptake, (E) tag synthesis, and (F) lipoprotein synthesis in dependence on plasma glucose concentrations. The blue curves depict control livers, while the red curves depict the livers of pregnant animals. The solid lines depict the mean values for each group, and the shaded areas depict the standard variation. Box plots on the right depict metabolic function under fed (12 mM plasma glucose) and fasted conditions (3 mM plasma glucose). The center lines represent the median, the boxes represent the interquartile range, and the whiskers are defined by values within 1.5 times the interquartile range. Outliers are represented as dots. Blue boxes show control livers and red boxes depict livers of pregnant animals. Red bars indicate significant differences assessed by a two-sided *t*-test with a p-value <0.01. (For interpretation of the references to color in this figure legend, the reader is referred to the Web version of this article.)

Fig. 3A–C shows that the overall capacities for fatty acid metabolism in the livers of pregnant rats are significantly decreased compared to control livers. The metabolism of fatty acids was examined by computing the cellular uptake rate of free fatty acids (FFAs) and the rate of central pathways of lipid metabolism in dependence on external FFA concentrations. In both groups, up to external FFA concentrations of ∼0.8 mM, the uptake rate of FFAs is increasing, while the uptake rate reaches saturation at higher plasma concentrations (Fig. 3A). The same saturation kinetics are observed for TAG synthesis (B) and lipoprotein secretion (C). While kinetics are similar, the livers of pregnant animals have decreased capacity for the uptake of fatty acids (A), the synthesis of TAG (B), and lipoprotein synthesis (C).

Fig. 3D–F shows the changes in fatty acid metabolism in pregnant rats in the physiological range from a fasted to a fed state. At fed state (i.e. 12 mM plasma glucose and ∼0.2 mM plasma fatty acid levels), the livers of pregnant animals have higher TAG synthesis rates (E) but similar fatty acid uptake rates (D) and lipoprotein synthesis rates (F) as control livers. In fasted states however (i.e. 3 mM plasma glucose and ∼1 mM plasma fatty acid concentrations), pregnant rats have significantly reduced fatty acid uptake rates (D), decreased TAG synthesis rates (E), and reduced production rates of lipoproteins (F).

Together these data show that livers of pregnant animals are characterized by reduced fatty acid metabolism concerning maximal capacities as well metabolic fluxes under physiological conditions and that these differences are most pronounced at high plasma fatty acid concentrations (fasted conditions).

### 2.4 Urea synthesis and ethanol detoxification

**Fig. 4:**
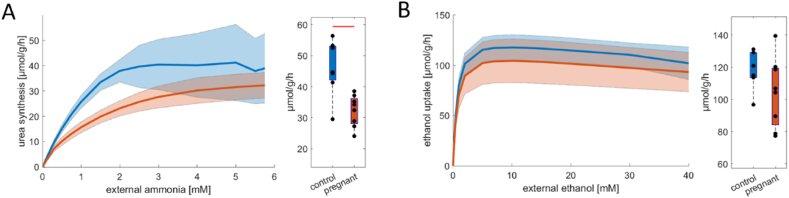
Comparison of ammonia and ethanol detoxification between control livers (n = 7) and livers of pregnant animals (n = 8). (A) Rate of urea production in dependence on plasma ammonia concentrations. (B) Rate of ethanol uptake in dependence on plasma ethanol concentrations. The solid lines depict mean values for each group, and the shaded areas depict standard variation. Box plots show maximal metabolic capacities. The center lines represent the median, the boxes represent the interquartile range, and the whiskers are defined by values within 1.5 times the interquartile range. Outliers are represented as dots. Blue boxes show control livers, while red boxes depict the livers of pregnant animals. Red bars indicate significant differences assessed by a two-sided *t*-test with a p-value <0.01. (For interpretation of the references to color in this figure legend, the reader is referred to the Web version of this article.)

Clearance of potentially harmful metabolites from the blood is one of the most important liver functions. We analyzed the capability of the livers of pregnant rats compared to control livers to produce urea and extract ethanol from the plasma. Fig. 4A depicts the capacity of urea production at increasing ammonia concentrations. Pregnant animals have a significantly reduced capacity for urea synthesis, especially at concentrations below 2 mM, which is well above the physiological range. When comparing ethanol detoxification capacity, there are no significant differences between the control livers and livers of pregnant animals (p-value = 0.2).

### 2.5 Classification

**Fig. 5:**
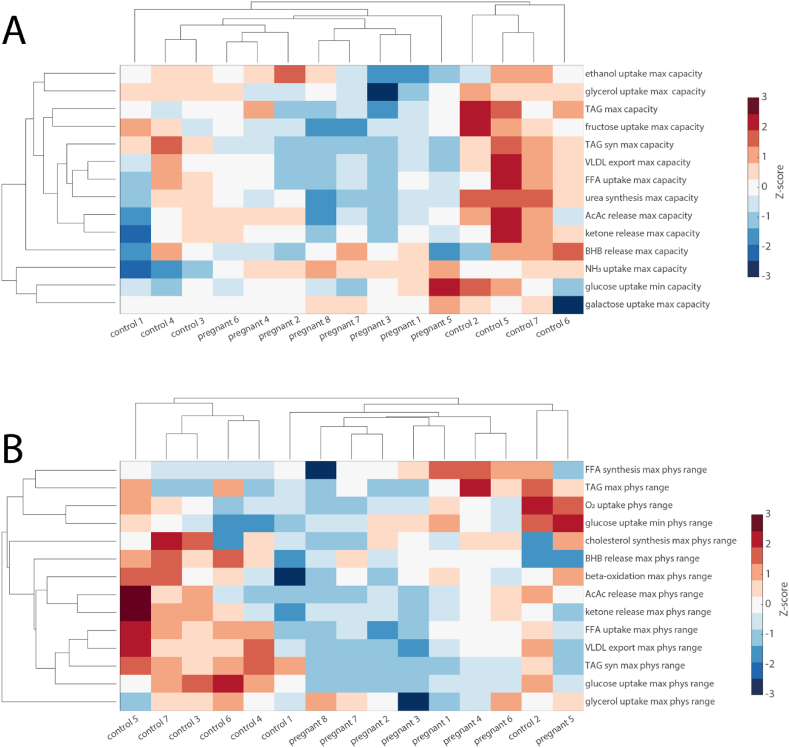
Unbiased clustering of maximal metabolic capacities (A) and maximal metabolic functions under physiological conditions (B). Values are normalized so that for each function the mean is 0 and the standard deviation is 1. Red and blue colors indicate increased and decreased metabolic function. Especially under physiological conditions, it can be seen that the livers of pregnant animals are characterized by an overall downregulation of metabolic capacities. (For interpretation of the references to color in this figure legend, the reader is referred to the Web version of this article.)

We have shown that there are significant differences in metabolic capacity between the livers of pregnant rats and control livers concerning carbohydrate metabolism, fatty acid metabolism, and detoxification capacities. However, not all metabolic functions are significantly different, raising the question of whether the differences can be used as discriminating markers. Therefore, we performed unbiased cluster analysis on the whole panel of investigated metabolic functions for the maximal capacities and in the metabolic functions under physiological conditions. As shown in Fig. 5A, for maximal capacities, the livers of pregnant rats form a cluster but that cluster is not perfectly separated from control livers. For metabolic functions under physiological conditions, depicted in Fig. 5B, the cluster is almost perfectly separated, except for one low-activity control sample. This shows that not only are the metabolic capacities and functions overall different for the two groups but they can be used as indicators for the individual livers. Overall, pregnant animals are characterized by a downregulation of the metabolic capability of the liver.

### 2.6 Proteomic signature

In general, metabolic functions are not attributable to single enzymes but are the result of the complex interactions in the metabolic network. Importantly, the relevance of a specific enzyme for a metabolic function might change dramatically depending on the metabolic situation [6]. However, it is still desirable to have a better understanding of the potential relevance of protein abundance changes for metabolic functionality. Therefore, to identify potential regulatory enzymes, we used a linear regression between the protein abundances for the different samples with the corresponding metabolic capacities. Furthermore, we checked whether the identified proteins were differentially abundant in the two groups.

For fatty acid metabolism, we identified 22 proteins, 18 of which exhibited significant differences between the groups. Of these, ten proteins were associated with fatty acid utilization, with seven showing significant differences, and ten were related to triglyceride (TAG) synthesis and storage, with nine showing significant differences. Additionally, two proteins involved in lipoprotein synthesis both showed significant differences. For carbohydrate metabolism, we identified 23 proteins, 19 of which were significantly different between the groups. Specifically, seven proteins were linked to fructose metabolism, with five showing significant differences, 13 were associated with glucose metabolism, with three showing significant differences, and three were involved in glycerol metabolism, with two showing significant differences. For urea synthesis, 15 proteins were identified of which nine were significantly reduced in pregnant animals, and for ethanol detoxification, two proteins were identified, both significantly different between the two groups. A complete list is given in the Supplementary Fig. S1.

### 2.7 Correlations between metabolic functions and biophysical properties

Imaging data showed that the livers of the pregnant group were significantly less viscous (higher *a* value) than those of the non-pregnant group (pregnant: 2.3 ± 0.4 m/s versus non-pregnant: 1.5 ± 0.1 m/s, p = 0.003). Similarly, a significant increase in water diffusivity (higher apparent diffusion coefficient (ADC) values) was observed in the pregnant group compared to the non-pregnant group (pregnant: 0.48e^−3^±0.02e^−3^ mm^2^/s versus non-pregnant: 0.42e^−3^±0.05e^−3^ mm^2^/s, p = 0.019). Shear wave speed *c* was not significantly different between the groups.

The liver samples included in the current study constituted a subgroup of a larger group as reported by Garczynska et al. [19], where histology analysis was conducted. This analysis based on H&E, Elastica van Gieson (EvG), and Ki-67 staining revealed the absence of ballooning, steatosis, and inflammation, as well as changes in collagen and elastic fibers in the animals. The observed alterations in biophysical properties were attributed to pregnancy-related hypertrophy and hyperproliferation of hepatocytes [19].

**Fig. 6:**
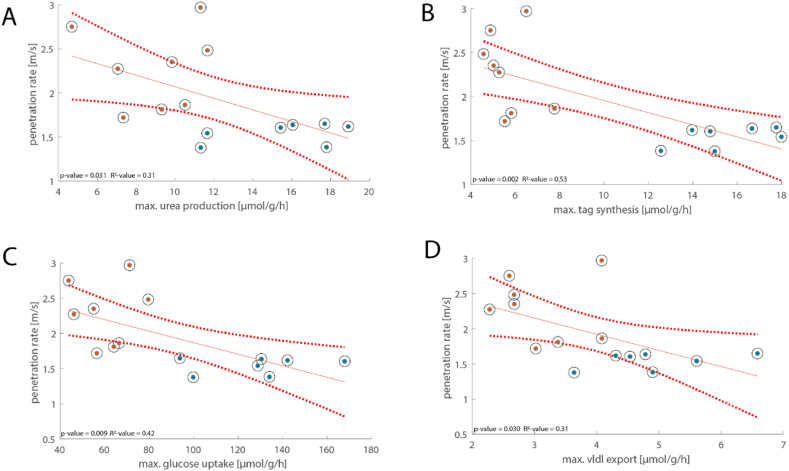
Significant correlations between penetration rate and metabolic capacities. (A) Urea production; (B) tag synthesis rate; (C) glycolysis rate; (D) vldl export. p-values according to the linear regression model with n = 15 (seven control livers, eight livers of pregnant animals) are given in each panel. Red solid lines indicate linear regression curves, and red dotted lines indicate 95 % confidence intervals of the linear model. Red dots depict individual values for livers of pregnant animals, while blue dots depict individual values for control livers. P- and R^2^-values are given in the panels. (For interpretation of the references to color in this figure legend, the reader is referred to the Web version of this article.)

We further explored the correlation between the imaging-based physical properties mentioned earlier and the metabolic functions. Based on the results from the correlation analysis, we found multiple significant correlations between the biophysical properties and metabolic functions. Using linear regression, five of the 14 maximal capacities (TAG synthesis (p = 0.003, R^2^ = 0.5), VLDL export (p = 0.048, R^2^ = 0.27), ammonia uptake (p = 0.023, R^2^ = 0.34), fructose utilization (p = 0.018, R^2^ = 0.36), and urea production (p = 0.046, R^2^ = 0.27)) were significantly associated with viscosity, and another two (fatty acid and glycerol uptake) had a tendency towards association (p-value <0.1). While no significant correlations could be found between the metabolic functions and liver stiffness, TAG content, β-hydroxybutyric acid production, and galactose uptake showed a tendency of association with liver stiffness (p-value <0.1). A tendency towards association has also been found between liver stiffness and fatty acid synthesis and glutamine exchange. When comparing viscoelastic properties with maximal metabolic functions under physiological conditions, significant correlations with maximal glucose uptake (p = 0.009, R^2^ = 0.42), VLDL export (p = 0.03, R^2^ = 0.31), TAG synthesis (p = 0.002, R^2^ = 0.53), and urea production (p = 0.031, R^2^ = 0.31) could be found (Fig. 6). Moreover, there was a tendency with fatty acid uptake (p-value <0.1). In addition to viscoelastic properties, water diffusion also showed a significant correlation with TAG synthesis, ammonia uptake, and glutamate exchange and has a tendency towards association with glucose uptake and VLDL export (p-value <0.1). The p- and R^2^-values for correlations between the biophysical properties and the different metabolic functions, as well as the corresponding linear regression fits, can be found in the Supplementary Figs. S2 and S3.

## 3 Discussion

The main objective of our work was to explore changes in metabolism in the liver of pregnant rats. Numerous studies have reported changes in lipid and carbohydrate metabolism by directly assessing fluxes or metabolite concentrations [[23], [24], [25]]. However, interpreting these data is cumbersome, as simultaneously occurring changes in enzyme activities, plasma nutrient and hormone composition, changes in liver mass, and contributions of peripheral tissue cannot be separated from changes in the hepatic metabolic activity itself. Furthermore, tracer characteristics and dynamics as well as spatial and temporal resolution might influence the determined rates. We chose an approach that combines experimentally determined protein abundance data (proteomics) with a molecular resolved kinetic model of central hepatic metabolism that has been validated based on metabolic experiments with hepatocytes, liver tissue, and perfused livers and has been applied to different problems like zonation, non-alcoholic steatohepatitis, and hepatocellular carcinoma [[26], [27], [28]]. This approach enabled us to overcome the conventional, ontology-based classification of metabolic pathway capacities by a quantitative assessment of hepatic functions in response to typical changes in blood plasma. During pregnancy, metabolism must change drastically to ensure an adequate supply of metabolites and cellular building blocks, and we found significant changes in carbohydrate metabolism, fatty acid metabolism, urea synthesis, and ethanol detoxification.

### 3.1 Carbohydrate metabolism

Our results show a drastic decrease in the utilization capacity of fructose and glycerol and no change in the capacity to utilize galactose and produce glucose. We also found a clear decrease in glycolysis under glucose-rich conditions (plasma glucose >8 mM). This implicates that during pregnancy, hepatic glucose metabolism is shifted towards decreased glucose utilization especially at hyperglycemic conditions, while changes in the physiological range of 6–8 mM are moderate. This finding is in line with the review by Lesser and Carpenter [11], where it was reported that glucose production increases with maternal body weight, such that glucose production per kilogram body weight does not change throughout pregnancy. Our analysis shows that the specific gluconeogenic capacity is not significantly altered in pregnant animals under physiological conditions but since total liver mass is significantly increased during pregnancy as reported by Garczynska et al. [19], total hepatic gluconeogenesis is significantly higher in pregnant animals than in the control group. Importantly, in line with other studies [29], glycogen storage is decreased in the livers of pregnant animals, which might potentially contribute to hyperglycemia in fasting periods during pregnancy.

### 3.2 Fatty acid metabolism

Our data show that the metabolic capacity for fatty acid uptake, synthesis and storage of TAG as well as lipoprotein synthesis are significantly diminished, especially under low glucose and high FFA conditions. At physiological conditions above 8 mM plasma glucose levels, however, the differences are small. This is especially noteworthy as increased plasma glucose levels are often observed during pregnancy [29]. This finding is also in line with the study by Wasfi et al. [30], where it was shown that hepatic uptake of oleic acid and TAG output were not significantly different per g liver in *ad* libitum-fed non-pregnant rats from rats on days 10, 15, and 20 of gestation. The total hepatic output of TAG was increased compared to that in the livers of non-pregnant rats simply due to a gain in liver mass, while no increase in hepatic TAG concentration was observed. This aligns with our data showing no difference in hepatic TAG content at physiological conditions. They also reported diminished ketogenesis during pregnancy, a trend we also find in our data but that is not significant due to a very high variability in ketogenesis in normal livers (data not shown). It is tempting to speculate on the proteomic origin of these changes. First, our data show a significant decrease in the fatty acid uptake transporter in the livers of pregnant rats by about 20 %. Furthermore, in pregnant animals, the decreased storage of TAG may be facilitated by significantly reduced amounts of perilipin-3, a protein shielding lipid droplets from lipolysis, thereby promoting TAG storage. Likewise, CGI58, a protein important for recruiting and activating lipases for TAG degradation, is significantly increased in pregnant rats promoting hepatic lipolysis. While no significant differences in CPT1 and CPT2, regulating the uptake of fatty acids into the mitochondria, were found between the two groups, we found a significant decrease in short and long-chain acyl-CoA dehydrogenase indicating a reduced capacity for mitochondrial β-oxidation. However, it is important to acknowledge that alterations in metabolic capacities, in general, originate from the coordinated regulation of multiple enzymes and that non-significant up or downregulation of multiple enzymes may result in significant changes on the functional level that cannot be assigned to specific individual enzymes.

### 3.3 Detoxification

Our data show that the livers of pregnant animals have a reduced capacity for urea synthesis. This is in line with the review by Kalhan [31], where it was reported that urea synthesis is significantly decreased as shown by stable-isotope-labelled tracers. While one can only speculate that the underlying reason is the salvage of nitrogen for protein synthesis during fetal development, the molecular basis for this adaptation is quite clear from our data. Most regulatory enzymes of the urea cycle like carbamoyl phosphate synthetase I, ornithine transporter, ornithine transcarbamylase, and argininosuccinate lyase are all consistently downregulated. Additionally, acylglutamate synthase, which synthesizes acyl-glutamate, a strong activator of the urea cycle [32] that is produced under high energy conditions, is severely decreased. This indicates that urea synthesis is deprioritized over other functions even if the energy supply is ample.

### 3.4 Association with biophysical properties

Recently, we reported that viscoelastic properties might be indicators of metabolic functions in healthy rabbit livers [20], and it was shown that biophysical characteristics including viscoelastic properties and water diffusivity differ between the livers of the non-pregnant and pregnant rats [19]. While it is desirable to have non-invasive biophysical imaging markers for predicting liver metabolic functions, the connections between the molecular profile determining metabolism and the liver's structure and composition, as indicated by biophysical parameters, remain largely unknown. In this study, we found various significant associations between biophysical properties and hepatic metabolic capacities and functions. Most noticeably, metabolic functions of fatty acid metabolism were found to be significantly associated with viscoelastic properties, especially with viscosity. A negative correlation between penetration rate (inverse viscosity) and hepatic fat fraction has been reported in pediatric patients with non-alcoholic fatty liver disease/steatohepatitis indicating that hepatic lipid deposition contributes to the wave attenuation properties of the liver [33]. As reported by Garczynska et al. [19], based on histopathology, there was no evidence of steatosis in the livers of pregnant rats used in this study. However, the alterations of fatty acid metabolism in pregnant animals found here may involve dysregulated lipid metabolic pathways, which led to minor retention of intrahepatic TAG. In the study by Garczynska et al. [19], the changes in imaging-based biophysical parameters, especially the changes in water diffusivity, were mostly attributed to pregnancy-related hepatocyte hypertrophy and hyperproliferation, which is a straightforward interpretation of the biophysical tissue behavior from a structural perspective. On the other hand, structural changes in the liver and alterations in the hepatic metabolism are not isolated events during pregnancy. It has been reported in the literature that pregnancy is associated with changes in metabolic parameters, such as hyperphagia, insulin, insulin-like growth factor, and growth hormone, which are likely to contribute to gestational hepatomegaly [[34], [35], [36]]. It is widely reported that metabolic regulation can remodel the cytoskeleton, thereby influencing cell behavior and the biophysical properties of the cell [37]. Thus, both the biophysical properties and the metabolic function of the liver were altered in the context of pregnancy. These structural and functional parameters were found to be correlated but the existence and extent of the causal relationship need to be further investigated preferably by studying disease models with defined metabolic disorders.

### 3.5 Limitations

There are a few important caveats that one should keep in mind when reading our data. First, our analysis uses the assumption that the circulating nutrient and hormone levels are identical in pregnant and non-pregnant animals, which most certainly is not the case. Unless we know the plasma nutrient and hormone composition, it is therefore not clear, which exact metabolic states should be compared. Second, we assume healthy relations between plasma glucose, fatty acid, and hormone levels, which might be dysregulated during pregnancy. Reduced hepatic insulin sensitivity, higher circulating levels of glucose, peripheral insulin resistance, and accompanied higher liberation of fatty acids from adipose tissue would all lead to altered fatty acid supply to the liver and therefore result in changes in fatty acid uptake, esterification as well as storage and lipoprotein release. Dysregulation of circulating nutrients and hormones is a common feature during pregnancy. Progressive decrease in peripheral insulin sensitivity [38], together with and mediated by a higher concentration of estrogen [39] are thought to be responsible for hypertriglyceridemia, impaired suppression of glucose production, and impaired stimulation of glucose utilization [3].

## 4 Conclusions

In summary, this study provides a comprehensive picture of hepatic metabolic remodeling during pregnancy in rats. We show that metabolic reprogramming occurs in carbohydrate metabolism, lipid metabolism, and ammonia detoxification. Importantly, the alterations are most pronounced under low glucose conditions, where the liver has significantly decreased metabolic capacities. This indicates that the liver maintains its homeostatic function under physiological conditions but might have compromised flexibility to respond to nutrient-demand mismatch e.g. in case of dietary restriction.

Interestingly, not all metabolic capacities are reduced. Pathways such as ketone body metabolism, gluconeogenesis, beta-oxidation, glycerol metabolism, fatty acid synthesis, and cholesterol metabolism remain relatively unchanged. Some of these adaptations may reflect alternative physiological demands during gestation, such as a decreased capacity for urea synthesis due to the increased need for ammonia in amino acid production.

However, it is also important to consider that pregnancy is associated with an increased risk of certain liver-related disorders, such as HELLP syndrome (hemolysis, elevated liver enzymes, and low platelets), pre-eclampsia, thrombotic thrombocytopenic purpura, and acute fatty liver of pregnancy. These conditions indicate that while the liver maintains its core functions during pregnancy, its ability to meet all demands under stress fully may be impaired. An increase in liver size during pregnancy [41] might therefore partially compensate for the loss of reserve capacity, allowing the liver to continue supporting both maternal and fetal needs despite additional challenges.

Moreover, this study has identified significant correlations between the biophysical properties and the metabolic capacities and functions of the liver during pregnancy, which may shed light on the complex interplay between function-structural regulations and the adaptation of the liver to physiological changes. Future studies will investigate changes in both the metabolism and biophysical properties of maternal rat livers under pregnancy-related complications, such as gestational diabetes and hypertension. These results will be compared to findings from normal pregnancies to substantiate the reproducibility of the current results.

The findings from the study on rats may have implications for understanding human pregnancy, as similar metabolic adaptations might occur in both species. During human pregnancy, the liver undergoes substantial changes in nutrient metabolism to support fetal development. Specifically, glucose metabolism is characterized by increased gluconeogenesis and reduced glycogen synthesis, comparable to the findings in the study [40,41]. Additionally, significant changes in whole-body fatty acid metabolism, including lipoprotein metabolism and circulating plasma lipids, are observed [42,43]. While these alterations can serve as markers for pregnancy-related liver diseases such as preeclampsia [44], the specific contribution of the liver is unclear.

Monitoring liver function and metabolic adaptations can aid in the early detection and management of these conditions, potentially improving outcomes for both mother and child. Especially non-invasive imaging techniques that detect changes in liver biophysical properties, as suggested by the rat study, could be valuable tools in prenatal care.

## 5 Material and methods

### 5.1 Sample preparation

All procedures involving animals were approved by the local authority (Reg.No. T0280/10, T0212/19) and were performed according to institutional guidelines. We used the ARRIVE reporting guidelines throughout the manuscript [45].

The liver samples were obtained from rats sourced from the animal pool in accordance with the Charité 3R (Replace, Reduce, Refine) principle. The size of the rat liver was appropriate for MRE and the remainder of *ex vivo* histological and biochemical analysis. Fifteen livers were harvested from young adult female Wistar rats (Forschungseinrichtungen für Experimentelle Medizin, FEM, Berlin, Germany; Janvier Labs, Le Genest-Saint-Isle, France) from which eight were sampled from pregnant rats (sacrificed on the 18th day of gestation) whereas seven were collected from non-pregnant rats. A subset of the specimens (six from pregnant animals, and six from non-pregnant animals) was studied using MRE and DWI, with sample size calculated based on the estimated effect size derived from changes in liver mass, biophysical properties, and associated metabolic changes during pregnancy, as reported by Garczynska et al. [19].

All animals were housed in the same animal facility under standardized conditions for a minimum of three days and were fed a normal chow diet provided by the facility. The ages of the pregnant and non-pregnant rats were 126 ± 22 days and 100 ± 7 days, respectively. For the pregnant group, the litter size was 16 ± 2. Rats were anesthetized with an overdose of isoflurane vapor and then decapitated using a rodent guillotine. The liver samples were collected in the morning between 9 a.m. and 12 p.m. The average weight of the livers from the non-pregnant and pregnant groups was 11.2 ± 1.1 g and 18.7 ± 1.2 g, respectively. The samples were obtained from either the left lateral or the right medial lobe. For each animal, a small piece of liver (0.013 ± 0.003 g) was cut from the fresh liver and snap-frozen in liquid nitrogen.

### 5.2 Proteome analysis

Fifteen snap-frozen liver tissue samples weighing between 10 and 41.4 mg were transferred to low protein binding tubes for proteomic analysis. Tissues were homogenized under denaturing conditions with a FastPrep (two times for 60 s, 4.5 m x s^−1^) in 1 mL of a fresh buffer containing 3 M guanidinium chloride (GdmCl), 10 mM tris(2-carboxyethyl)phosphine, 20 mM chloroacetamide, and 100 mM Tris-HCl pH 8.5. Lysates were boiled at 95 °C for 10 min in a thermal shaker, followed by sonication for 10 min, and centrifuged at 16,000 rcf for 10 min at 4 °C. The supernatant was transferred into new protein low binding tubes (Eppendorf, Germany). 30 μg protein per sample were diluted to 1 M GdmCl by adding 10 % acetonitrile and 25 mM Tris, 8.5 pH, followed by a Lys C digestion (Roche, Basel, Switzerland; enzyme to protein ratio 1:50, MS-grade) at 37 °C for 2 h. This was followed by another dilution to 0.5 M GdmCl and tryptic digestion (Roche, 1:50) at 37 °C, at 800 rpm, and overnight. Subsequently, peptides were desalted with C18 columns and reconstituted in 2 % formic acid in water and further separated into five fractions by strong cation exchange chromatography (SCX, 3M Purification, Meriden, CT). Eluates were first dried in a SpeedVac, then dissolved in 5 % acetonitrile and 2 % formic acid in water, briefly vortexed, and sonicated in a water bath for 30 s before injection to nano-LC-MS/MS.

### 5.3 LC-MS/MS instrument settings for shotgun proteome profiling and data analysis

Liquid Chromatography with tandem mass spectrometry (LC-MS/MS) was carried out by nanoflow reverse-phase liquid chromatography (Dionex Ultimate 3000, Thermo Scientific) coupled online to a Q-Exactive HF Orbitrap mass spectrometer (Thermo Scientific), as reported previously [46]. Briefly, the LC separation was performed using a PicoFrit analytical column (75 μm ID × 50 cm long, 15 μm Tip ID; New Objectives, Woburn, MA) in-house packed with 3-μm C18 resin (Reprosil-AQ Pur, Dr. Maisch, Ammerbuch, Germany). Peptides were eluted using a gradient from 3.8 to 38 % solvent B in solvent A over 120 min at a 266 nL per minute flow rate. Solvent A was 0.1 % formic acid and solvent B was 79.9 % acetonitrile, 20 % H_2_O, and 0.1 % formic acid. For the IP samples, a 1-h gradient was used. Nanoelectrospray was generated by applying 3.5 kV. A cycle of one full Fourier transformation scan mass spectrum (300−1750 *m*/*z*, resolution of 60,000 at m/z 200, automatic gain control (AGC) target 1 × 10^6^) was followed by 12 data-dependent MS/MS scans (resolution of 30,000, AGC target 5 × 10^5^) with a normalized collision energy of 25 eV.

Raw MS data were processed with MaxQuant software (v 1.6.10.43) and searched against the Rattus norvegicus proteome database UniProtKB (UP000002494) with 29,942 entries, released in June 2020. Parameters of MaxQuant database searching were a false discovery rate (FDR) of 0.01 for proteins and peptides, cysteine carbamidomethylation was set as fixed modification, while N-terminal acetylation and methionine oxidation were set as variable modifications.

### 5.4 Evaluation of metabolic capacities

We used HEPATOKIN1 [15] in combination with a detailed model of lipid droplet metabolism [47], as previously used by Berndt et al. [27], to evaluate the functional implications of protein abundance changes observed during late pregnancy in the liver. The model comprises the central hepatic metabolic pathways of glycolysis, gluconeogenesis, glycogen synthesis, glycogenolysis, fructose metabolism, galactose metabolism, the creatine phosphate/ATP shuttle system, the pentose phosphate cycle composed of the oxidative and non-oxidative branch, the citric acid cycle, the malate aspartate redox shuttle, the glycerol-3-phosphate shuttle, the mitochondrial respiratory chain, the beta-oxidation of fatty acids, fatty acid synthesis, ketone body synthesis, cholesterol synthesis, TAG synthesis and degradation, the synthesis and hydrolysis of TAG, the synthesis and export of VLDL, the urea cycle, the metabolism of the amino acids serine, alanine, glutamate, glutamine, and aspartate, and ethanol metabolism. The model contains the key electrophysiological process of the inner mitochondrial membrane including the mitochondrial membrane potential, mitochondrial ion homeostasis, and the generation and utilization of the proton motive force described by kinetic equations of the Goldman-Hodgkin-Katz type [48]. The metabolic model is coupled to a phenomenological model of hormonal signaling by glucagon and insulin affecting the short-term regulation of metabolic enzymes by reversible phosphorylation. As plasma glucose, fatty acid, insulin, and glucagon concentrations are not independent but tightly linked through the pancreatic and adipose tissue-controlled release of hormones and fatty acids, we used phenomenological transfer functions describing the dependence of the plasma concentrations of insulin and glucagon and non-esterified fatty acids (NEFA) on plasma glucose levels. Plasma concentrations of insulin and glucagon were directly translated into the phosphorylation state of interconvertible enzymes by a phenomenological function described by Bulik et al. [6]. Varying external plasma glucose concentrations from 3 mM (corresponding to a severely fasted state with high glucagon, low insulin, and high fatty acid levels) to 12 mM (corresponding to a well-fed state with low glucagon, high insulin, and low fatty acid levels) enables us to evaluate hepatic metabolic functions in the whole physiological range between the fasted and fed state. The model describes the uptake, metabolization, and generation of glucose, fructose, galactose, pyruvate, lactate, glycerol, ammonia, serine, alanine, glutamate, glutamine, fatty acids, ethanol, acetate, urea, acetoacetate, β-hydroxybutyrate, oxygen, and VLDL particles [15]. For a detailed description see Berndt et al., 2018 and 2021 [15,28]. The enzymatic rate equations and external conditions for each load characteristic are given by Berndt et al. [15].

### 5.5 Parametrization of individualized kinetic metabolic models of livers of pregnant and control rats

We used the protein abundance data obtained from LC-MS/MS analysis to generate individual instantiations of the metabolic model describing the liver of each animal. For this purpose, the maximal activities $v_{\max}$ of enzymes and transporters $E_{a}$ for each animal were computed according to $v_{\max}^{E_{a}}=v_{\max}^{E_{ref}}\frac{E_{a}}{E_{ref}}$, where $E_{a}$ and $E_{ref}$ denote the label-free quantification intensities for protein E in the animal a and the reference liver, respectively. As a reference, we used the mean label-free quantification intensities of protein E in control livers. The maximal activity $v_{\max}^{E_{ref}}$ of enzyme E for the reference livers was assumed to be identical to the generic model HEPATOKIN1 that has been fitted to experimental data of fluxes and metabolites obtained in perfused whole livers, isolated cells, or tissue sections derived from whole livers. All rate equations and the $v_{\max}^{E_{ref}}$ values can be found in Berndt et al. [15].

### 5.6 Magnetic resonance elastography (MRE) and diffusion weighted imaging (DWI)

As detailed by Garczynska et al. [19], liver tissue slices (approximately 20 mm in height, 5–8 mm in width) were freshly harvested from the mice. These slices were slid into a glass tube with a diameter of 7.5 mm. The tube was inserted into a 0.5-T compact MRI scanner (Pure Devices GmbH, Würzburg, Germany) for both MRE and DWI measurements. MRE used mechanical vibration of 800 Hz while DWI was performed with seven b-values (50, 175, 300, 425, 550, 675, and 800 s/mm^2^). Further details on the imaging parameters for both MRE and DWI were described by Garczynska et al. [19]. Liver stiffness (shear wave speed, *c* in m/s) and inverse viscosity (penetration rate, *a* in m/s) were quantified using an analytical solution for cylindrical waves, as described by Braun et al. [49]. Water diffusivity (represented by the apparent diffusion coefficient, ADC in μm^2^/s) was quantified by mono-exponential fitting and linear regression analysis applied to images at all seven b-values.

### 5.7 Statistical analysis

Data were checked for normality by one-sample Kolmogorov-Smirnov test. Significant differences between groups were assessed by a two-sided *t*-test for normal distributed group values, otherwise, the Wilcoxon signed-ranked test was used. Statistical analysis and cluster analysis were done with MATLAB Release 2023b (The MathWorks, Inc., Natick, MA, USA) with the bioinformatics toolbox.

Statistical analysis of proteomic data was performed by a two‐sample t‐test with Benjamini–Hochberg correction for multiple testing. Significantly regulated proteins in the livers of pregnant animals compared to controls are listed in Supplementary Table S1.

For comprehensive proteome data analyses, gene set enrichment analysis using GSEA software (v4.3.3) with the Kegg database was applied. GSEA standard settings were used, except that the permutation setting was changed to ‘gene sets’ as recommended for less than ten samples per group. The cut‐off for significantly regulated pathways was set to ≤0.05 p‐value and ≤0.05FDR.

Principal component analysis was performed to reduce the dimensionality of the proteomic dataset. The MATLAB ‘pca’ function was employed on the dataset. The results were used to identify the principal components that capture the most significant variance within the data.

Hierarchical clustering analysis was conducted using MATLAB's ‘clustergram’ function to explore patterns in the proteomic dataset. The function was applied to the dataset to generate a heatmap with hierarchical clustering of both rows (proteins) and columns (samples). This visualization enabled the identification of clusters of proteins with similar expression profiles and patterns of coexpression across samples.

To assess the relationship between the metabolic functions and biophysical properties, a linear regression model was employed using MATLAB's ‘fitlm’ function. This function fits a linear model to the data by estimating the coefficients through least squares minimization. The statistical significance of the predictors was determined using p-values, with a threshold set at 0.05.

## CRediT authorship contribution statement

**Jing Guo:** Writing – original draft, Funding acquisition, Data curation, Conceptualization. **Karolina Krehl:** Writing – review & editing, Data curation. **Yasmine Safraou:** Writing – review & editing, Validation, Investigation. **Iwona Wallach:** Writing – review & editing, Data curation. **Jürgen Braun:** Writing – review & editing, Supervision, Methodology, Funding acquisition. **David Meierhofer:** Writing – review & editing, Methodology, Investigation. **Ingolf Sack:** Writing – review & editing, Funding acquisition, Formal analysis. **Nikolaus Berndt:** Writing – original draft, Visualization, Software, Methodology, Funding acquisition, Formal analysis, Data curation, Conceptualization.

## Ethics statement

All procedures involving animals were approved by the Landesamt für Gesundheit und Soziales (LAGeSo), Berlin (Reg.No. T0280/10, T0212/19).

## Data and code availability

The MS proteomics data have been deposited to the ProteomeXchange Consortium via the PRIDE [50] partner repository with the dataset identifier PXD050758. The metabolic models used have been published previously [15,47].

## Declaration of generative AI and AI-assisted technologies in the writing process

During the preparation of this work, the authors used ChatGP 4o (OpenAI, San Francisco, California, U.S.) in order to refine the language. After using this tool, the authors reviewed and edited the content as needed and take full responsibility for the content of the publication.

## Declaration of competing interest

The authors declare that they have no known competing financial interests or personal relationships that could have appeared to influence the work reported in this paper.
