# Supplementary material for: Pregnancy alters fatty acid metabolism, glucose regulation, and detoxification of the liver in synchrony with biomechanical property changes
Source: Heliyon. 2024 Oct 22;10(20):e39674. doi: 10.1016/j.heliyon.2024.e39674 (PMC11538949; doi:10.1016/j.heliyon.2024.e39674)

Pregnancy alters fatty acid metabolism, glucose regulation, and detoxification of the liver in synchrony with biomechanical property changes

Jing Guo, Karolina Krehl, Yasmine Safraou, Iwona Wallach, Jürgen Braun, David Meierhofer, Ingolf Sack, Nikolaus Berndt

***Corresponding author**

Nikolaus Berndt

German Institute of Human Nutrition Potsdam-Rehbruecke (DIfE), Dept. of Molecular Toxicology

Arthur-Scheunert-Allee 114-116 | 14558 Nuthetal, Germany

Phone: + 49 33 200 88 - 2365 | e-mail: nikolaus.berndt@dife.de

Table of Contents

Supplementary Tables 3
Table S1 3
Supplementary Figures 5
Supplementary Fig. S1 Capacity-Protein Correlations 5
(A) Ethanol detoxification 5
(B) Fatty acid utilization 6
(C) Fatty acid utilization 6
(D) Fatty acid utilization 7
(E) Fatty acid utilization 7
(F) Triacylglycerol (TAG) synthesis 8
(G) TAG synthesis 8
(H) TAG synthesis 9
(I) TAG synthesis 9
(J) Lipoprotein synthesis 10
(K) Fructose metabolism 10
(L) Fructose metabolism 11
(M) Fructose metabolism 11
(N) Glycerol uptake 12
(O) Urea synthesis 12
(P) Urea synthesis 13
(Q) Urea synthesis 13
(R) Urea synthesis 14
(S) Urea synthesis 14

Supplementary Fig. S2 Correlations between the biophysical properties and the metabolic functions under physiological conditions 15
(A) Urea production 15
(B) TAG synthesis 16
(C) TAG storage 16
(D) Ammonia uptake 17
(E) Ketone body production 17
(F) Gluconeogenesis 18
(G) Glucose uptake 18
(H) Glutamate exchange 19
(I) Glutamine exchange 19
(J) Fatty acid uptake 20
(K) Fatty acid synthesis 20
(L) Cholesterol synthesis 21
(M) Beta-oxidation 21
(N) Very-low-density lipoprotein (VLDL) production 22
(O) Acetoacetate production 22
(P) Beta-hydroxybutyrate production 23
Supplementary Fig. S3 Correlations between the biophysical properties and the metabolic functions – Maximal capacities 23
(A) Ethanol uptake 24
(B) Acetoacetate production 24
(C) Beta-hydroxybutyrate production 25
(D) Fatty acid uptake 25
(E) Ketone body uptake 26
(F) TAG storage 26
(G) TAG synthesis 27
(H) VLDL production 27
(I) Fructose uptake 28
(J) Galactose uptake 28
(K) Glycerol uptake 29
(L) Gluconeogenesis 29
(M) Ammonia uptake 30
(N) Urea production 30

Supplementary Tables

Table S1

A complete list of differentially regulated proteins in the livers of pregnant rats compared to non-pregnant animals.

Protein	Adjusted p-value	Fold change
Gpt	4,46967E-10	-1,85948486
Cyp2e1	1,32176E-08	-1,0114077
Nucb2	3,65243E-08	1,132442328
Itih4	4,63822E-08	1,249052557
RGD1564515	9,59995E-08	2,339801658
Txndc5	1,74787E-07	1,121084912
Ghr	2,94642E-07	1,343226079
Pon1	4,17597E-07	-1,38903531
Cyp2c23	5,30031E-07	-1,10320973
Tdo2	9,14619E-07	1,474159785
Rbp1	9,36038E-07	1,266827059
Erlec1	1,10911E-06	1,079916281
Serpind1	1,18975E-06	1,008261131
Ecm1	1,28618E-06	-1,31912475
Enpep	2,0253E-06	-1,29411848
Cars	2,06098E-06	1,254863877
Bckdk	2,82213E-06	1,235274054
Sec23b	3,23682E-06	1,084036568
Tat	3,3351E-06	1,279116213
Ostc	4,26001E-06	1,125806441
Ifih1	6,03487E-06	1,125725115
Aspg	6,09719E-06	-1,17021056
Hmox1	6,30661E-06	1,006455132
A1m	6,80242E-06	1,087892235
Sgk2	7,12439E-06	1,327655031
Ppib	8,03619E-06	1,06109836
Cyp1a2	9,5515E-06	-1,30528927
LOC100912604	1,01749E-05	1,016769168
Inf2	1,18268E-05	1,469347455
Fmo5	1,42266E-05	-1,18739966
Eps8l2	1,48168E-05	-2,04410127
Prlr	1,71578E-05	1,553524253
Ggcx	2,04904E-05	1,120959113
Fads1	3,80956E-05	1,214052443
Abhd5	3,97749E-05	1,71297585
C6	4,98737E-05	1,482249706
Ube2o	5,58361E-05	1,622300209
Cyp2t1	5,74916E-05	-1,13056325
Igh-1a	6,7617E-05	-1,51571952
Abcg2	7,41811E-05	1,46453023

Ftl1;RGD1566189;LOC100359668;LOC100362384;Ftl1l1;RGD1560687	0,000148493	-1,0335802
Bche	0,000221714	1,310290253
Golgb1	0,000250418	1,103053677
Agpat3	0,000274668	1,661563613
Fmo5	0,000295493	-1,40200063
Adh6	0,000380683	-1,05364866
Dsg2	0,000468263	-1,19116236
Fads2	0,000481895	1,108761339
Ugt2a3	0,00054402	-2,00660312
Akr7a3	0,00066194	-1,33059231
S100a8	0,000878277	1,268765973
LOC100363239	0,000944143	1,385511651
MGC108823;LOC100910979	0,000988818	1,351181267
S100a9	0,001221064	1,423756794
Orm1	0,001487371	-1,1070007
Hsd11b1	0,001533249	1,441809185
C1galt1	0,001754962	1,067373375
Cyp8b1	0,001768257	-1,24699418
Mpa2l;LOC498435	0,002032028	1,335368925
Pcdh1	0,002139198	-1,2811971
Ldhb	0,002222678	1,066203188
Prlr	0,002437701	1,742206037
Hgfac	0,00453066	1,026119511
Acsm2	0,005065323	-1,72677103
Fen1	0,007361157	1,062455975
Akr1c12	0,007613422	-1,57195659
Fabp7	0,008811216	1,094570845
Stmn1	0,008944547	1,65704866
Plin2	0,010097998	1,397405962
Fndc3b	0,01256438	1,056082676
Abcb1a;Abcb1b	0,013695036	-1,08843465
Csad	0,015532174	-1,12339177
Nlrc3	0,015889107	-1,19178763
Anp32e	0,017480082	1,723592694
Minpp1;RGD1564801	0,026309831	1,103557489
Fnbp1l	0,026664322	1,47323413
Ifit3	0,028436515	1,055663884
Tp53i11	0,03013711	2,047423124
Crip1	0,030346386	1,780689281
Abcg3l1;Abcg3l3	0,034612388	1,045874544
Tmem205	0,041742616	1,125653348
Crbn	0,044181673	1,035625052
LOC501233	0,047281001	-1,17871307

Supplementary Figures

Supplementary Fig. S1 Capacity-Protein Correlations

Significantly regulated functional proteins were determined by a linear regression model assessing the association between maximal metabolic capacities and the abundance of proteins belonging to the respective pathway. Red dotted lines indicate 95% confidence intervals of the linear model given by the solid red line. Dots represent values of protein abundance and associated metabolic function for individual samples (blue: control liver, n= 6; orange: liver of pregnant animals, n = 6). The level of significance was p <0.05. For the significantly associated functional proteins, the differences between groups were assessed by a two-sided t-test.

For fatty acid metabolism, we identified 22 proteins of which 18 were significantly different between the groups (fatty acid utilization (10/7), triacylglycerol (TAG) synthesis and storage (10/9), and lipoprotein synthesis (2/2)). For carbohydrate metabolism, we identified 23 proteins of which 19 were significantly different between the groups (fructose (7/5), glucose (13/3), and glycerol (3/2)). For urea synthesis, 15 proteins were identified of which nine were significantly reduced in livers of pregnant rats, and for ethanol detoxification, two proteins were identified, both significantly different between the two groups. All significantly associated proteins and the respective metabolic functions are depicted below. The upper panels show the linear regression model, p-values are given in the lower right corner. Lower panels show box plots of protein abundance levels for control livers and livers of pregnant animals. The center lines represent the median, the boxes represent the interquartile range, and the whiskers are defined by values within 1.5 times the interquartile range. Individual values for the different samples are represented as black dots. Red and black bars indicate significant differences with p-values <0.01 and <0.05, respectively.

(A) Ethanol detoxification

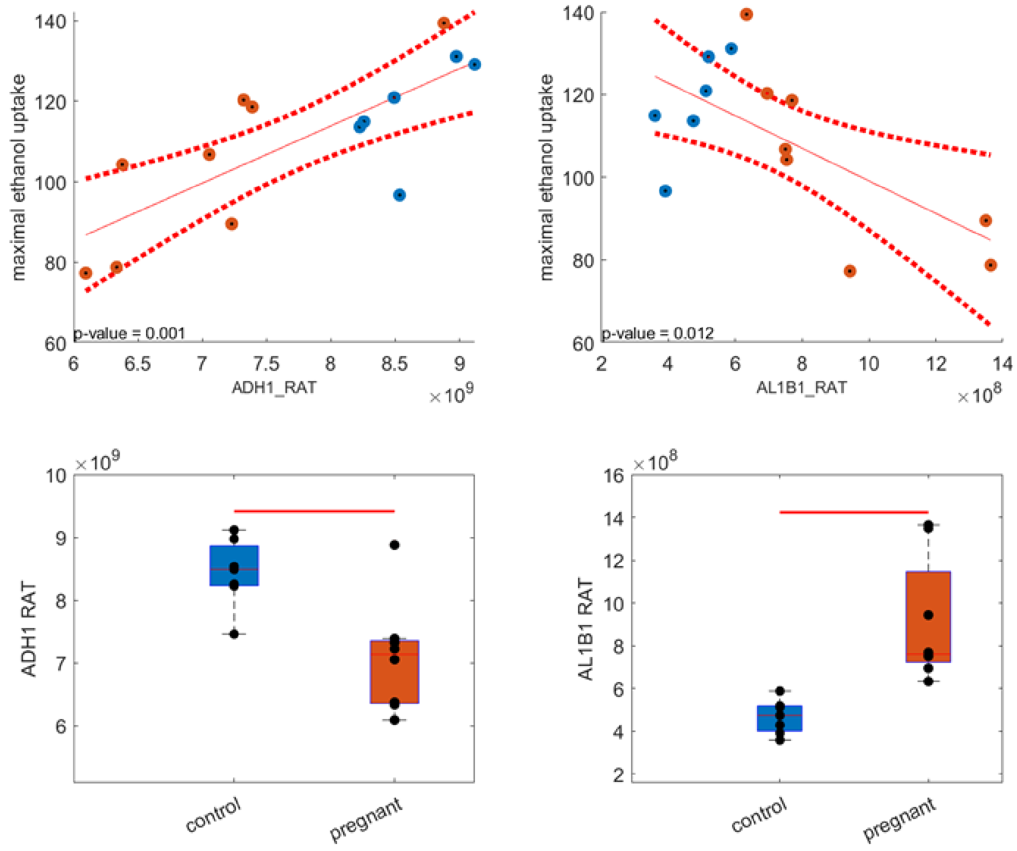

(B) Fatty acid utilization

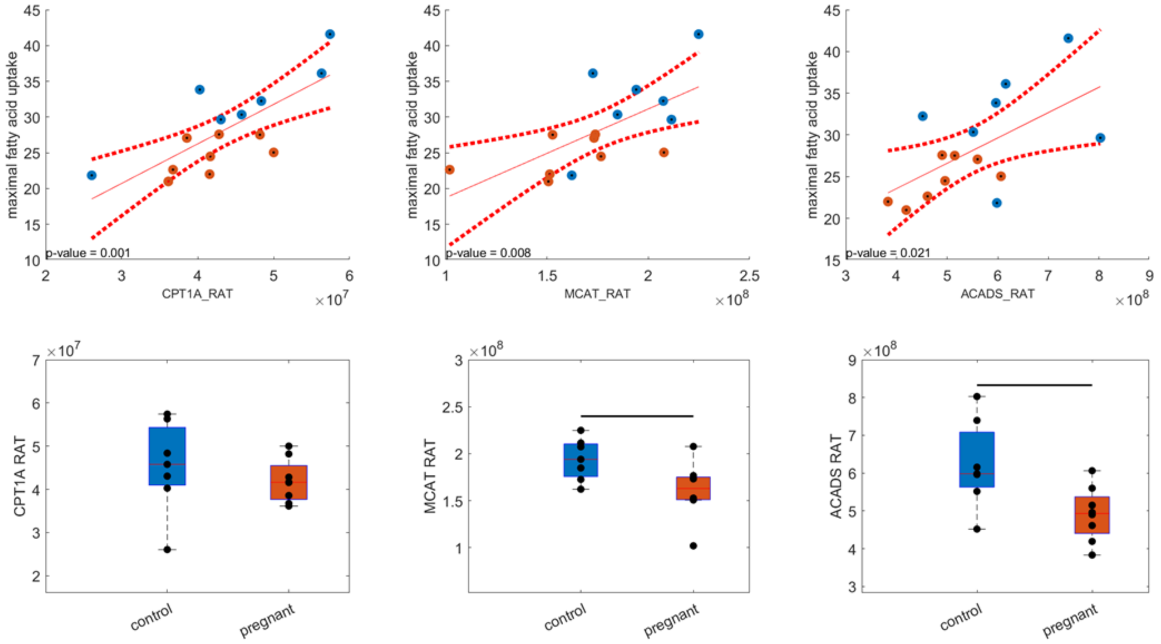

(C) Fatty acid utilization

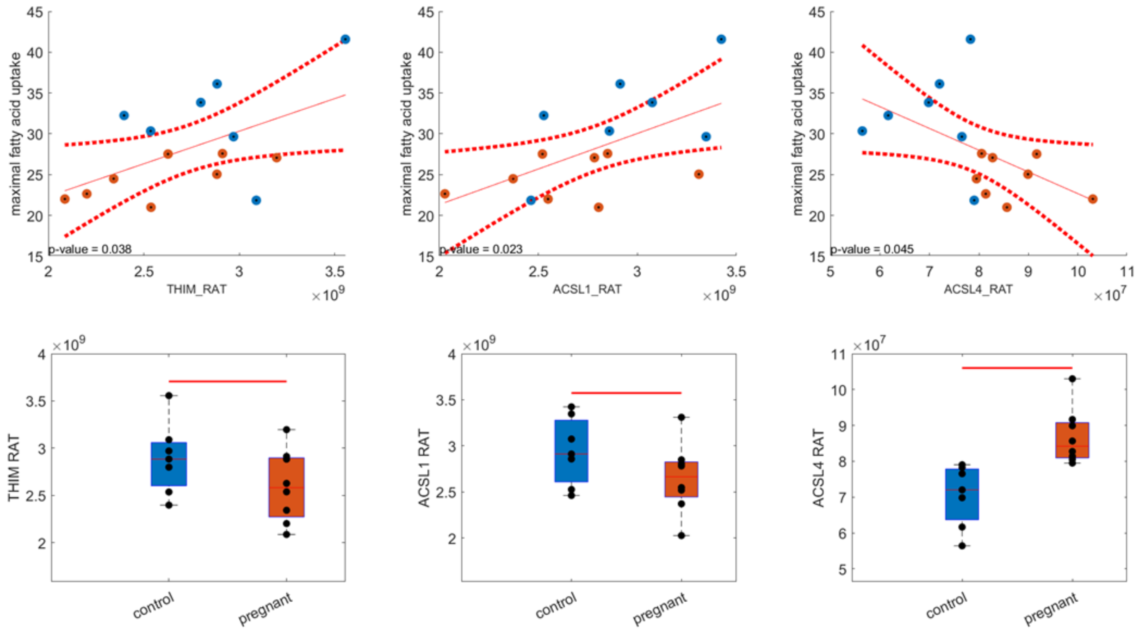

(D) Fatty acid utilization

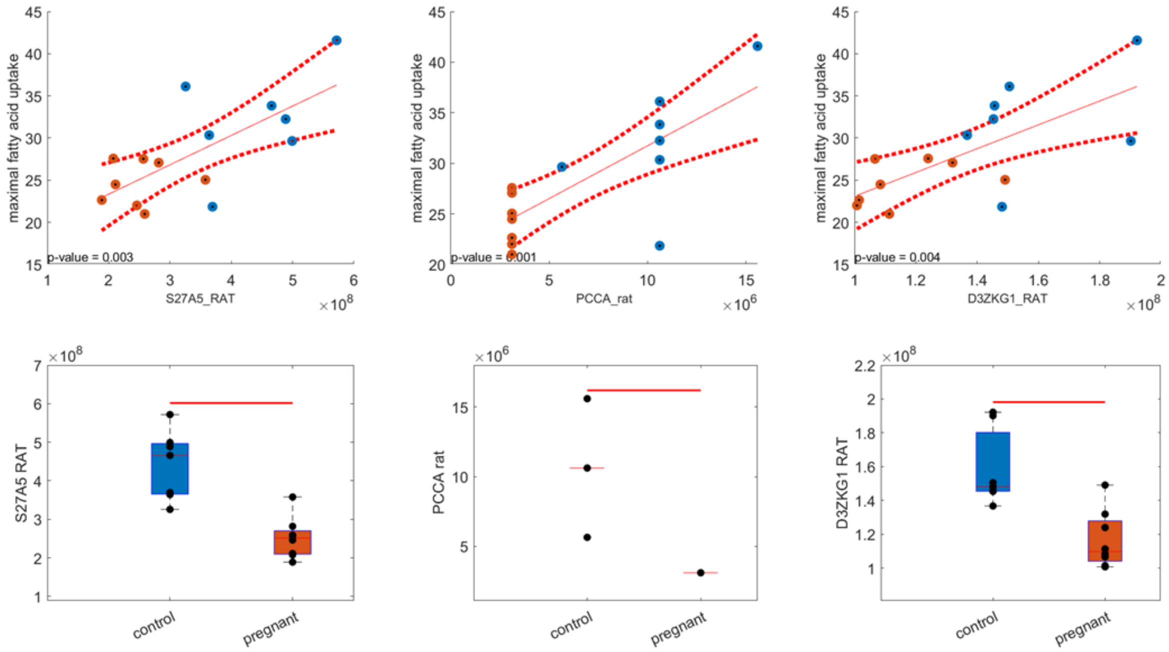

(E) Fatty acid utilization

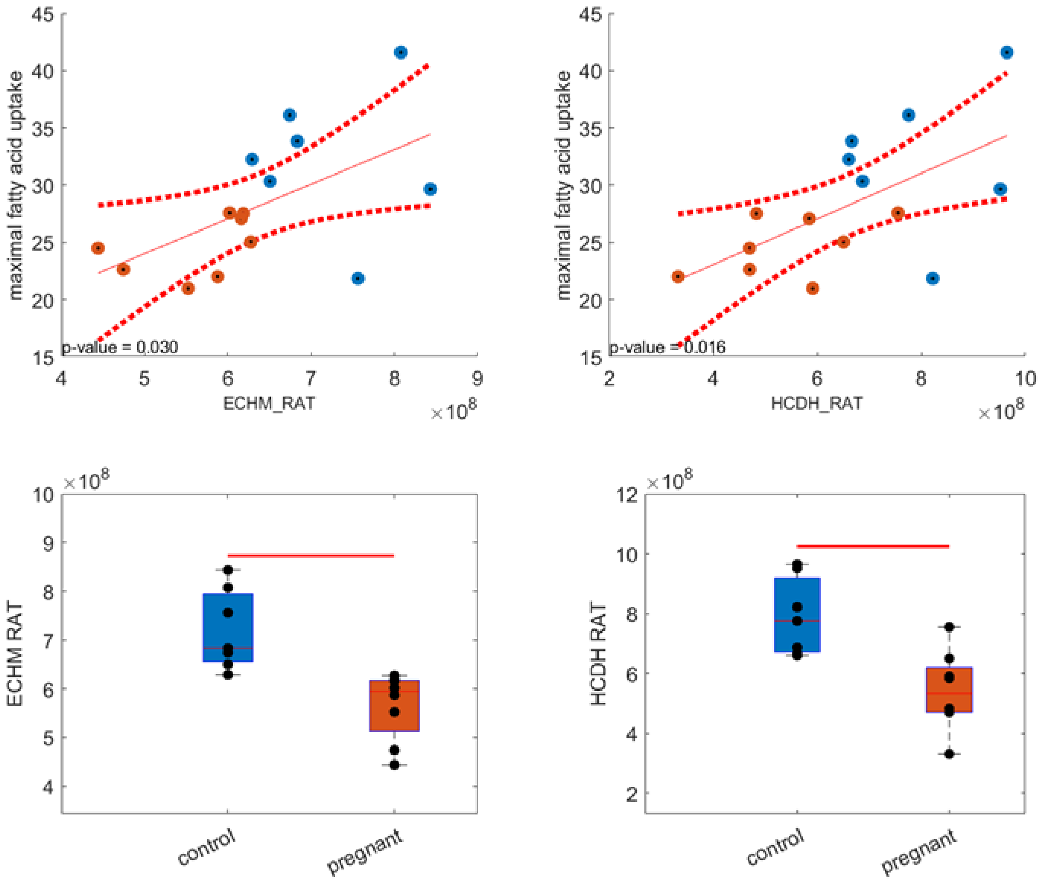

(F) Triacylglycerol (TAG) synthesis

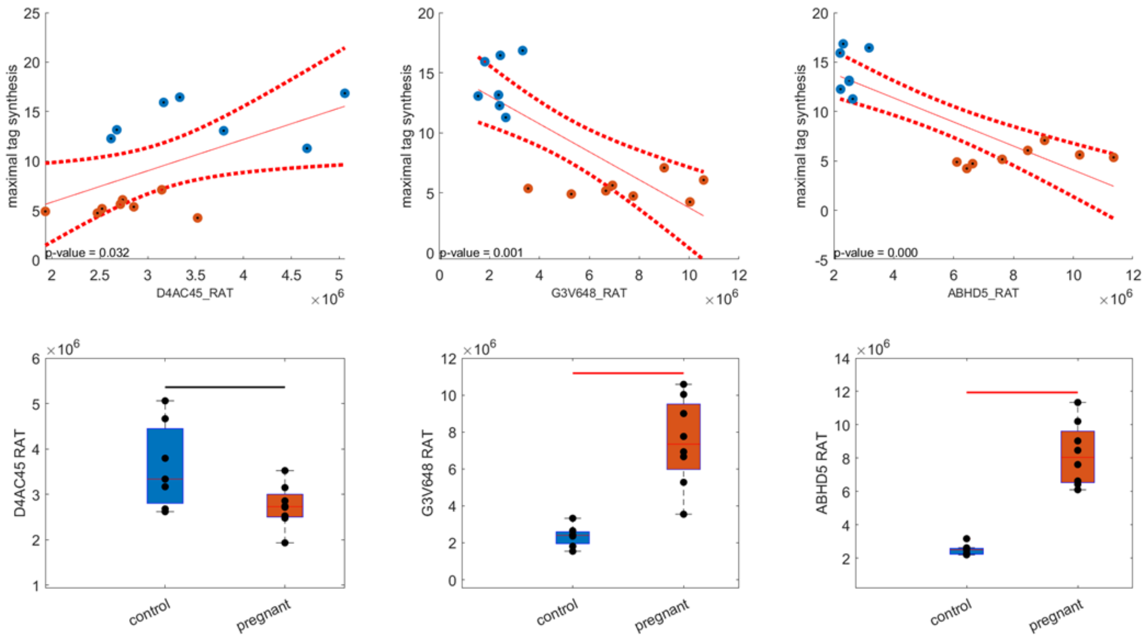

(G) TAG synthesis

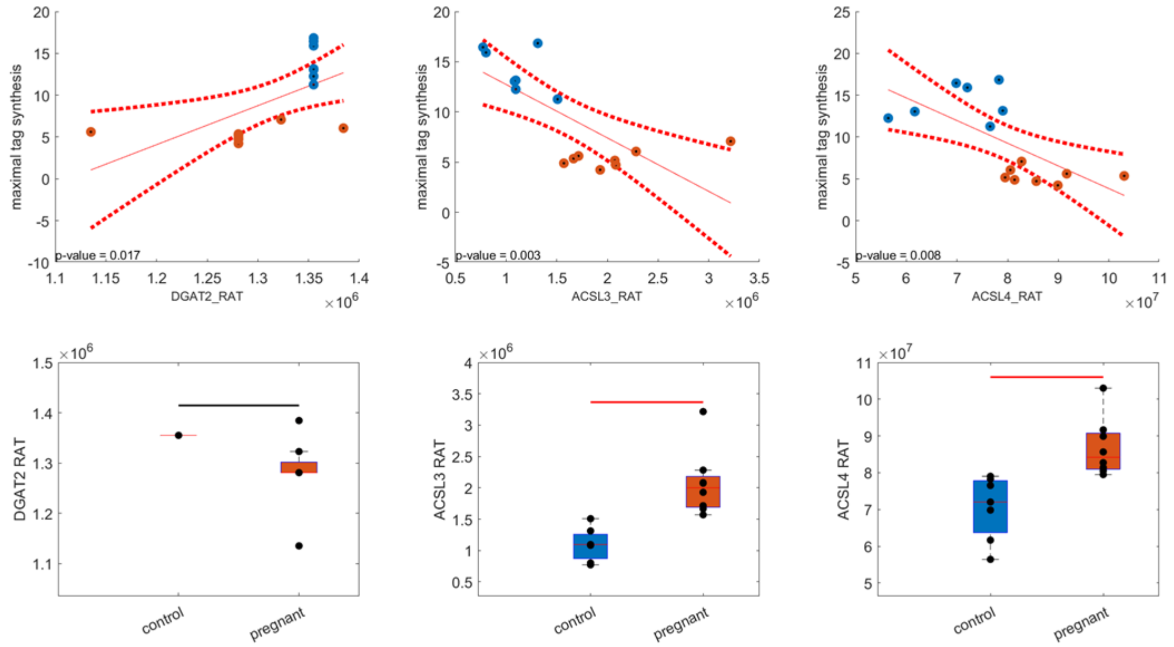

(H) TAG synthesis

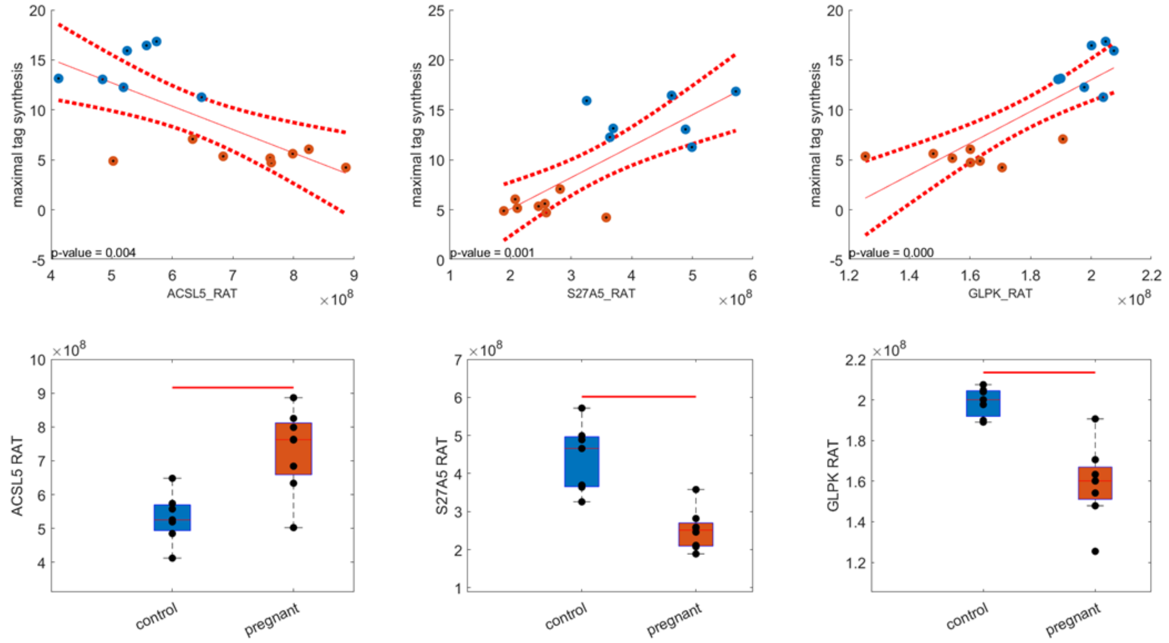

(I) TAG synthesis

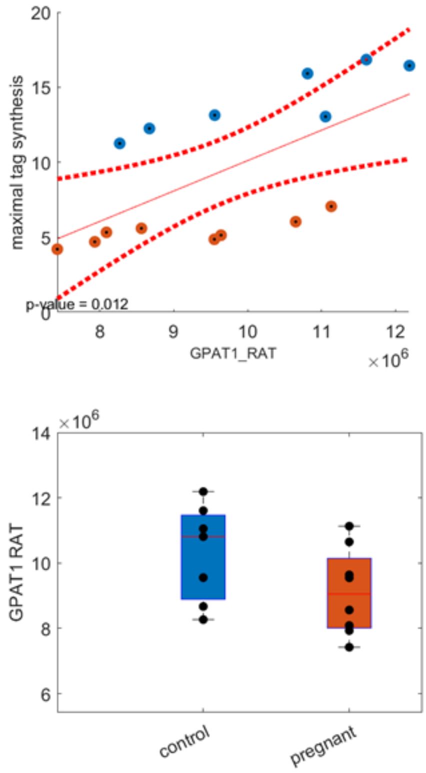

(J) Lipoprotein synthesis

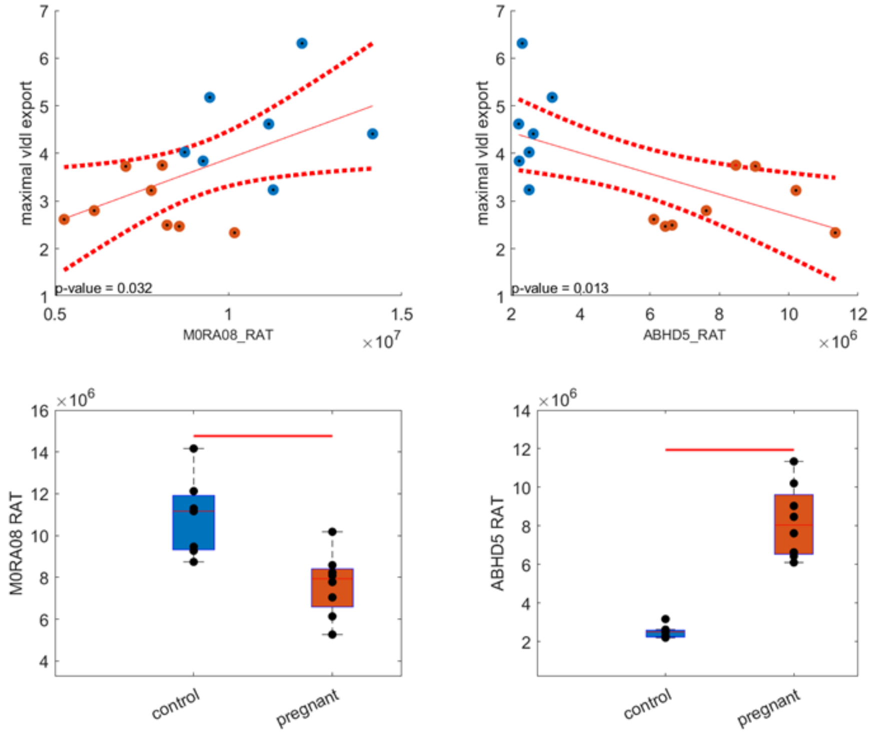

(K) Fructose metabolism

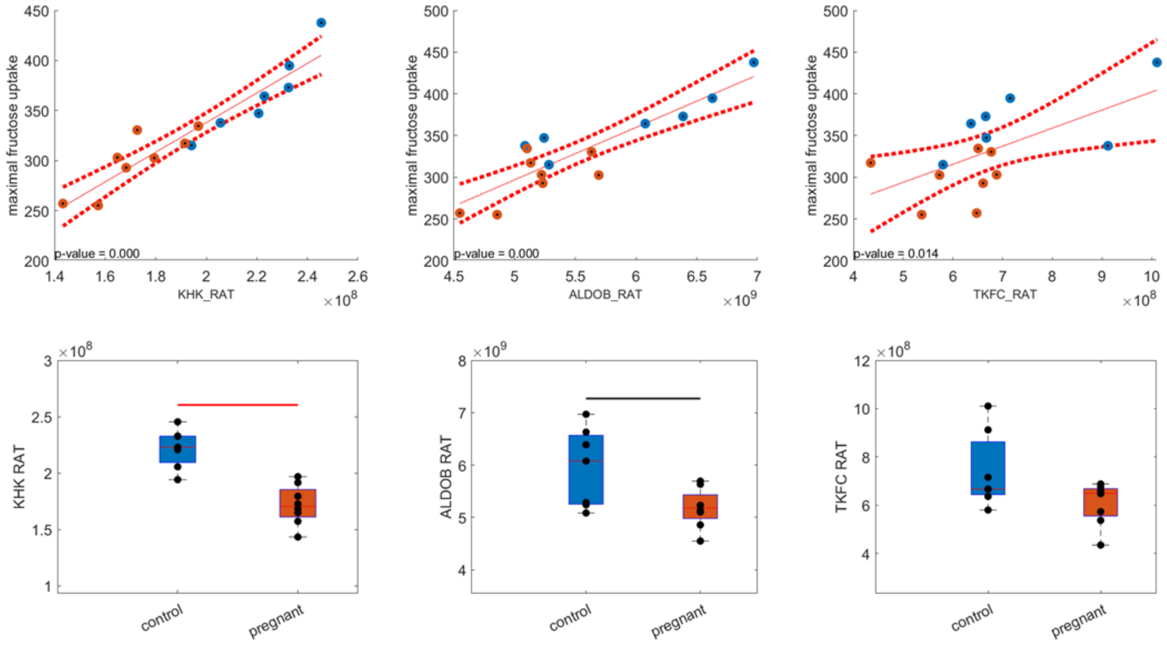

(L) Fructose metabolism

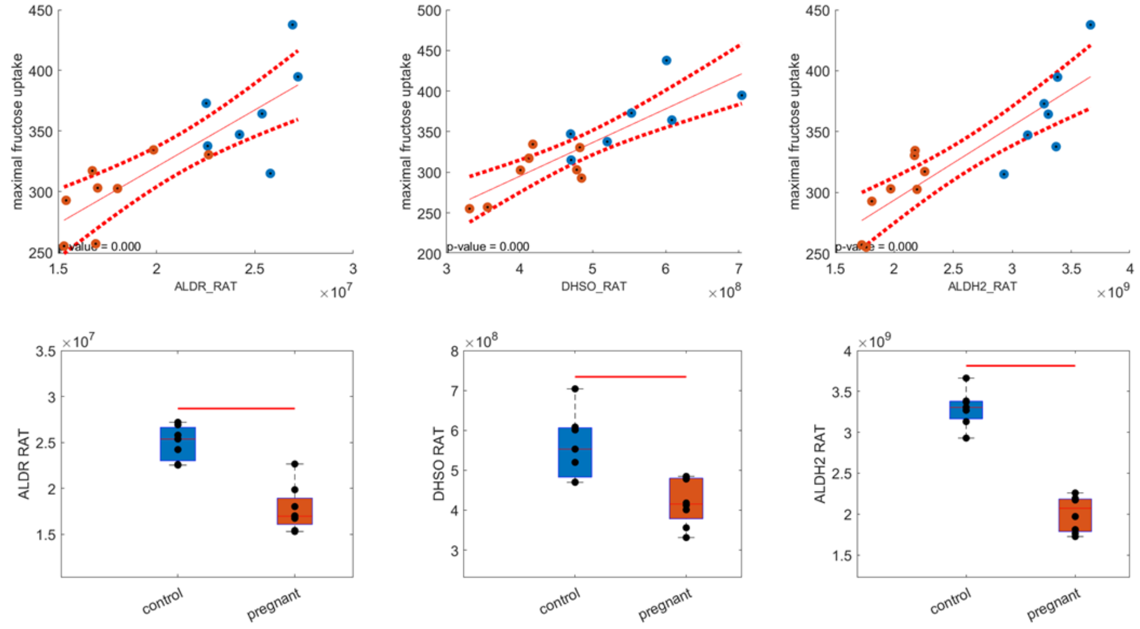

(M) Fructose metabolism

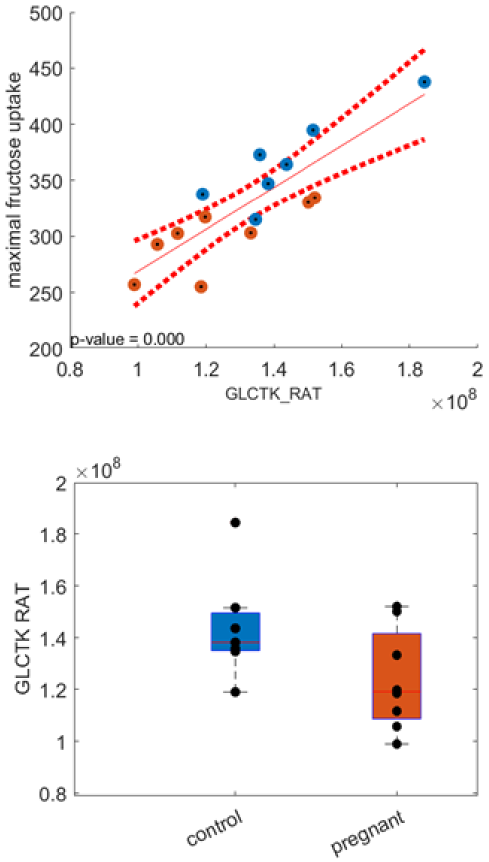

(N) Glycerol uptake

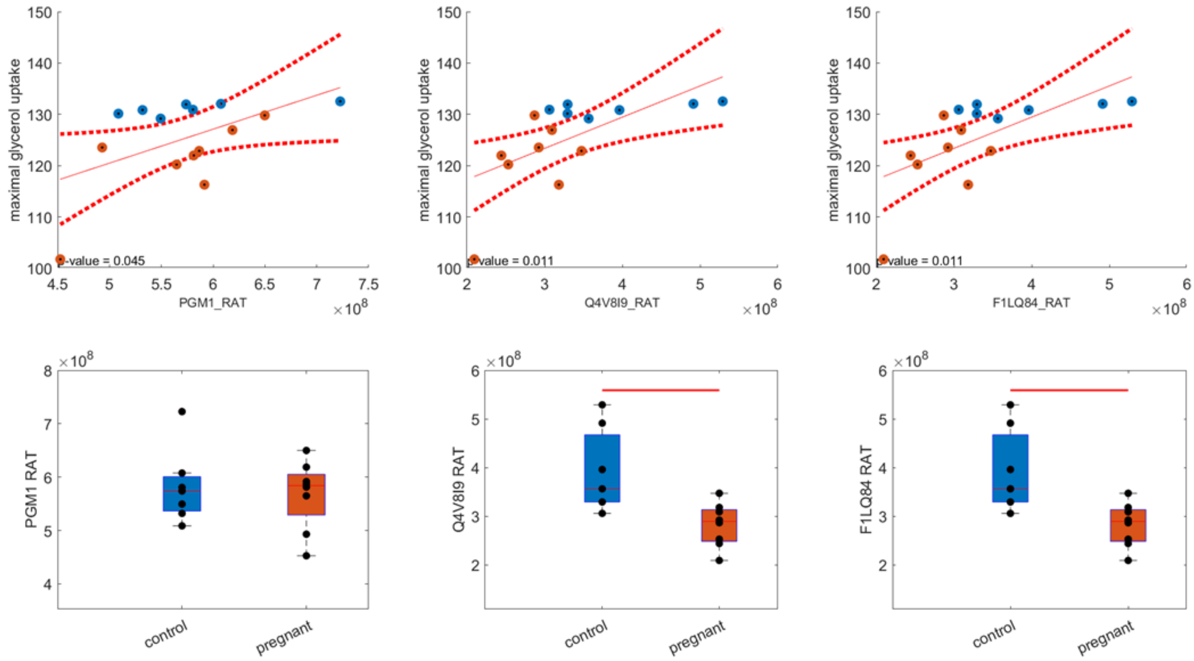

(O) Urea synthesis

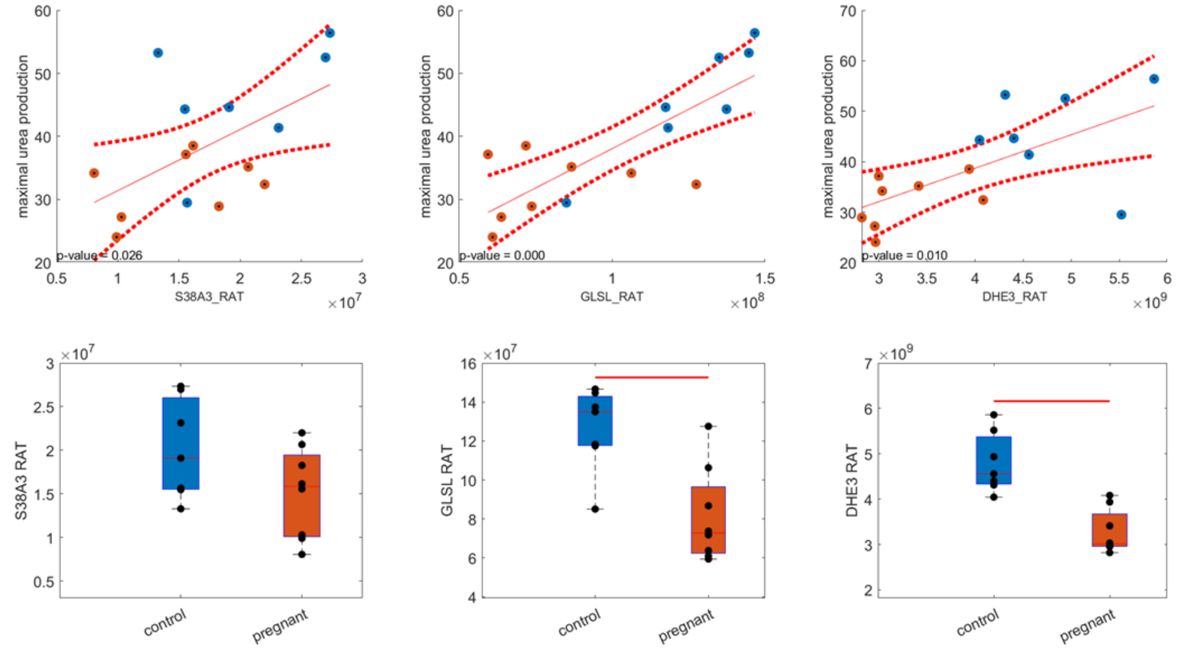

(P) Urea synthesis

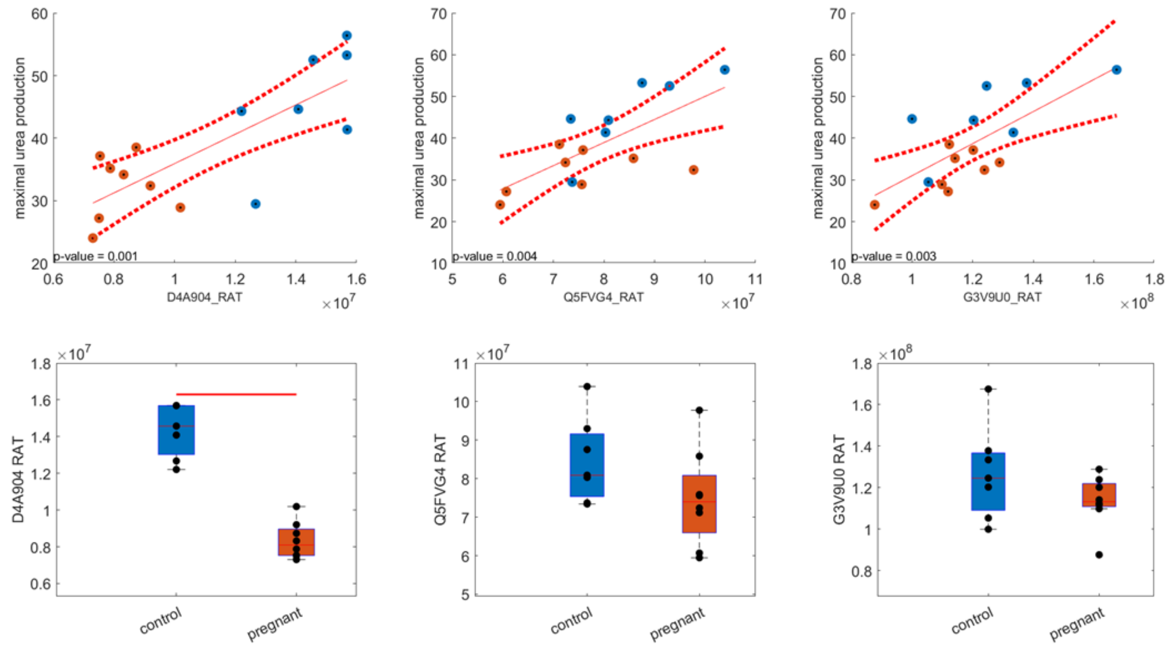

(Q) Urea synthesis

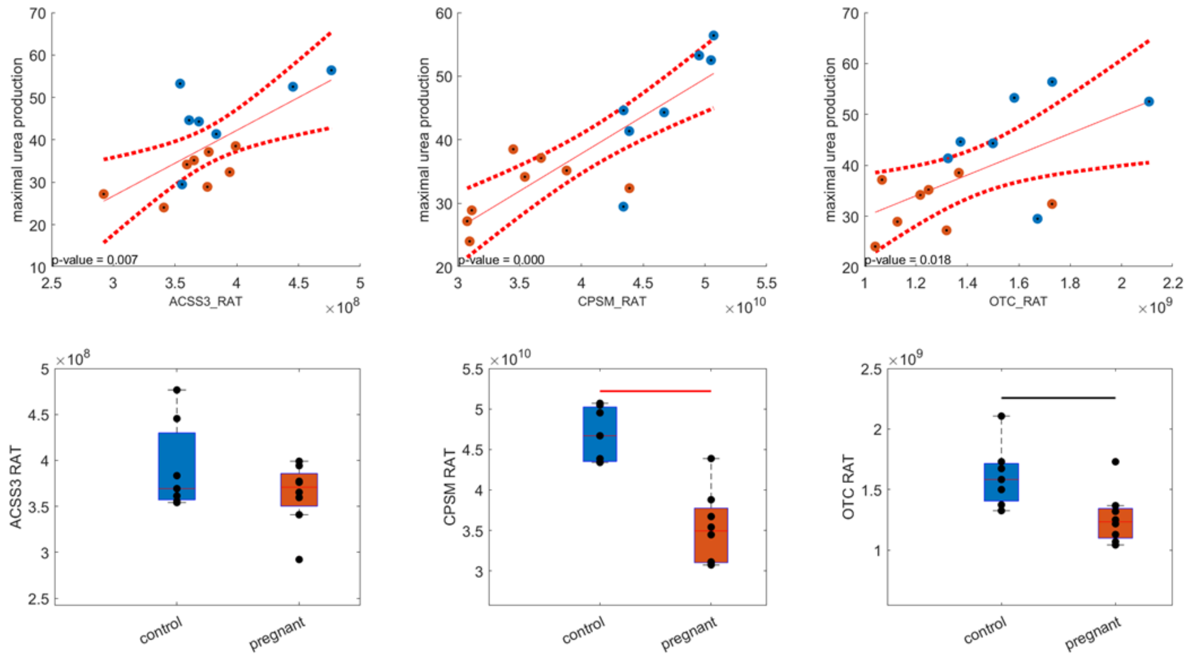

(R) Urea synthesis

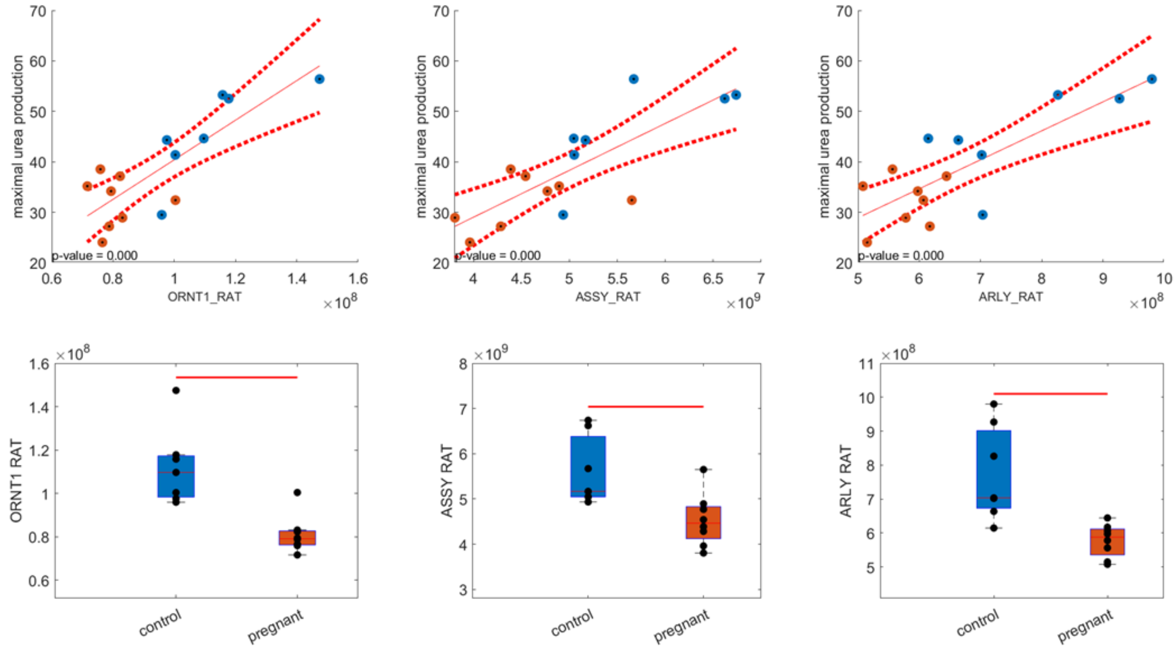

(S) Urea synthesis

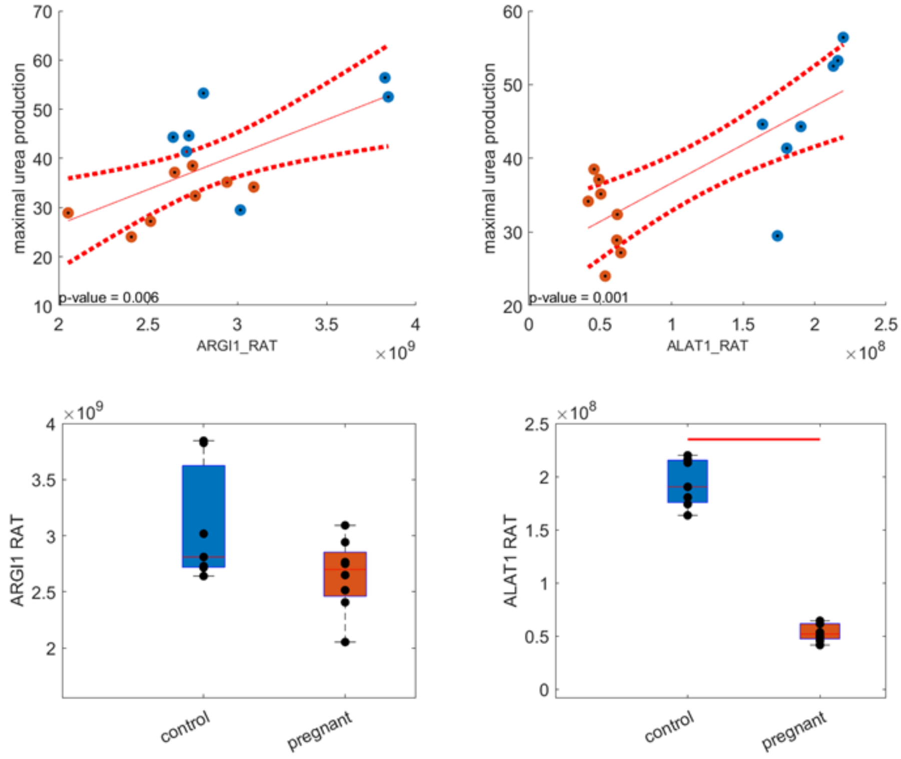

Supplementary Fig. S2 Correlations between the biophysical properties and the metabolic functions under physiological conditions

The association between visco-elastic properties and the different metabolic functions was determined by a linear regression model. Linear regression fit and associated p-values for the association between visco-elastic properties (*c* in m/s, shear wave speed; *a* in m/s, penetration rate; and ADC in 10^{-3} mm^2/s, apparent diffusion coefficient) and the different metabolic functions are given below.

The differences in the metabolic functions between the control livers (n = 6) and the livers of pregnant animals (n = 6) are given by the box plots in the first panels. The center lines represent the median, the boxes represent the interquartile range, and the whiskers are defined by values within 1.5 times the interquartile range. Individual values for the different samples are represented as black dots. P-values are given above the brackets. Red and black brackets indicate significances with p-values <0.01 and <0.05, respectively. Orange and yellow brackets indicate a tendency with p-values <0.1 and <0.25, respectively. The 2nd to 4th panels show a linear regression model between the metabolic function and the visco-elastic properties. Red dotted lines indicate 95% confidence intervals of the linear model given by the solid red lines. Dots represent values of protein abundances and associated metabolic functions for individual samples (blue: control liver, orange: liver of pregnant rats). P- and R^2-values for linear regression are given in the lower right corner.

(A) Urea production

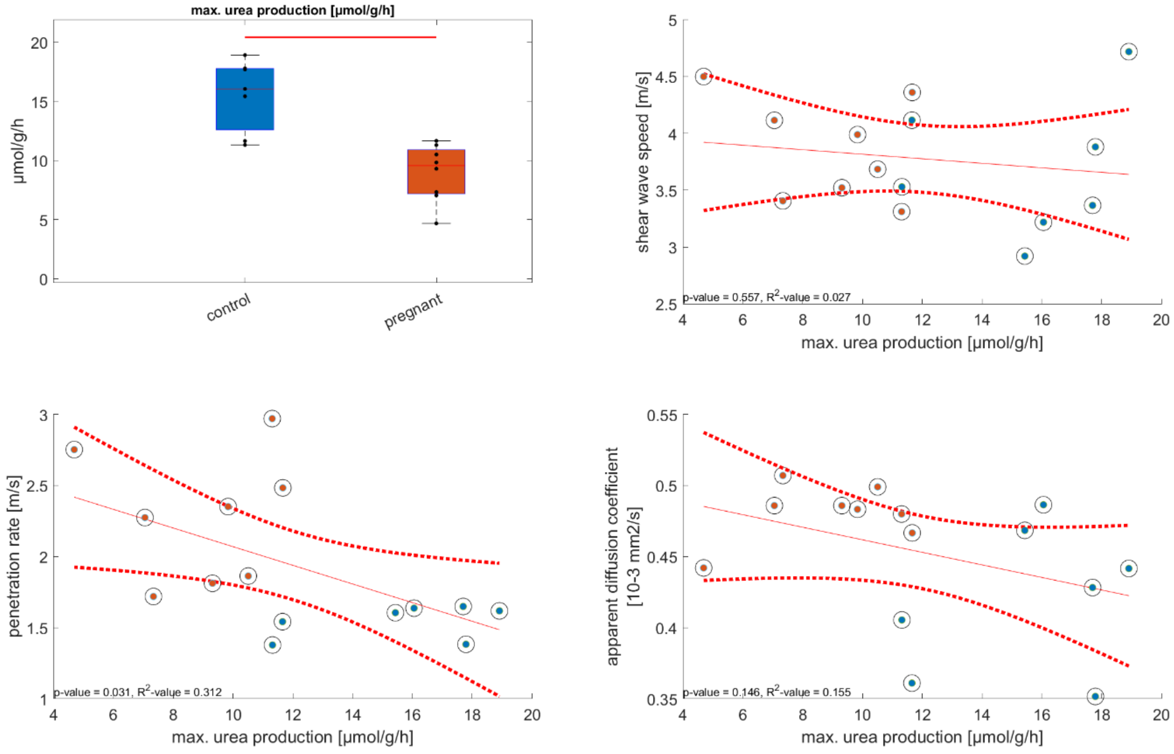

(B) TAG synthesis

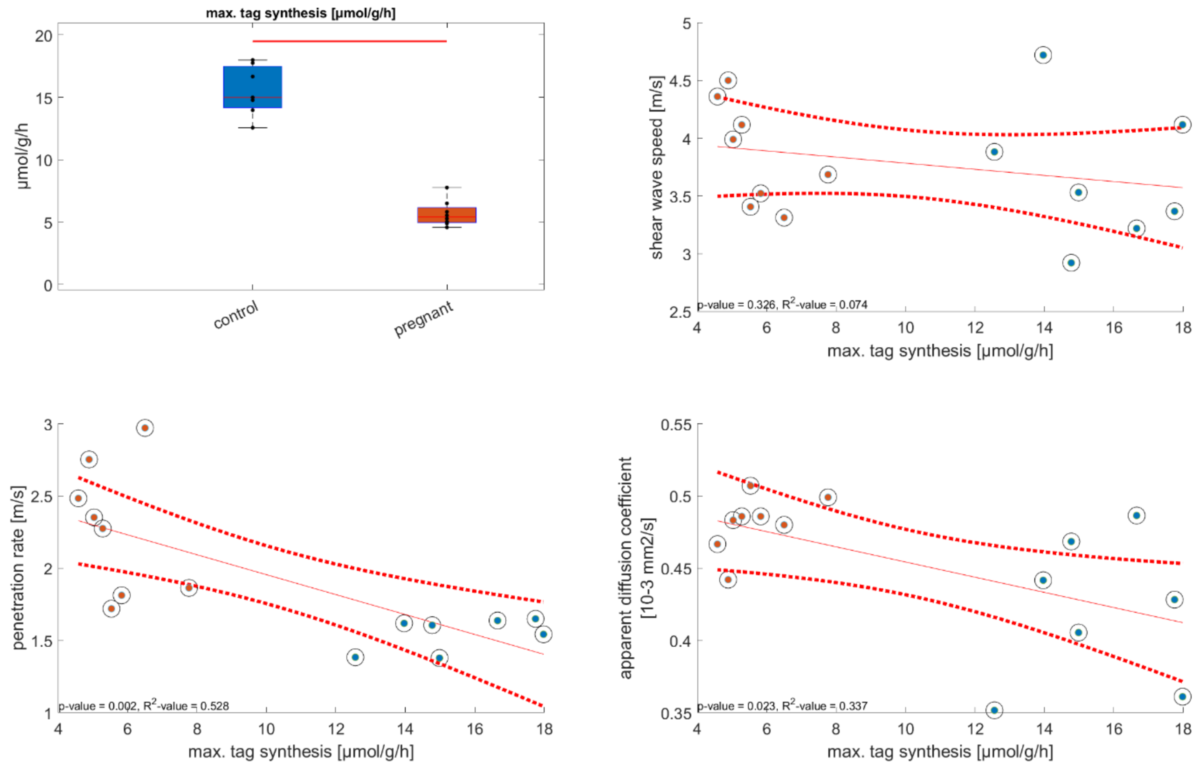

(C) TAG storage

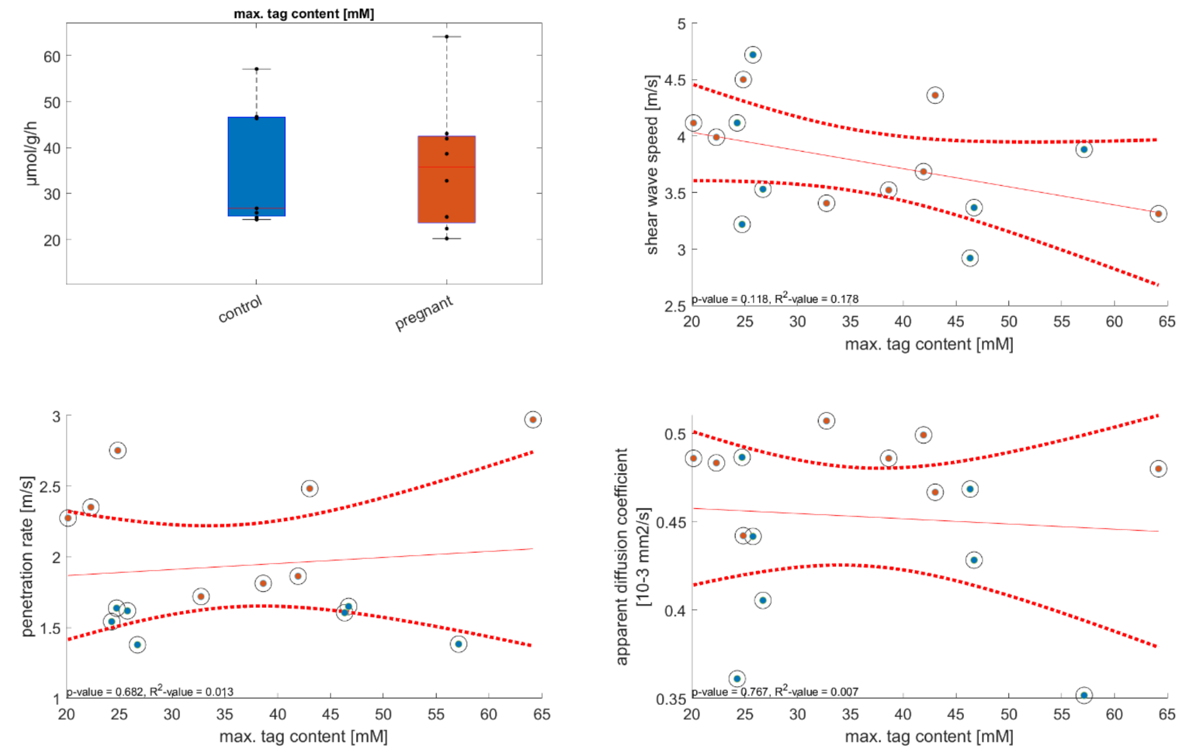

(D) Ammonia uptake

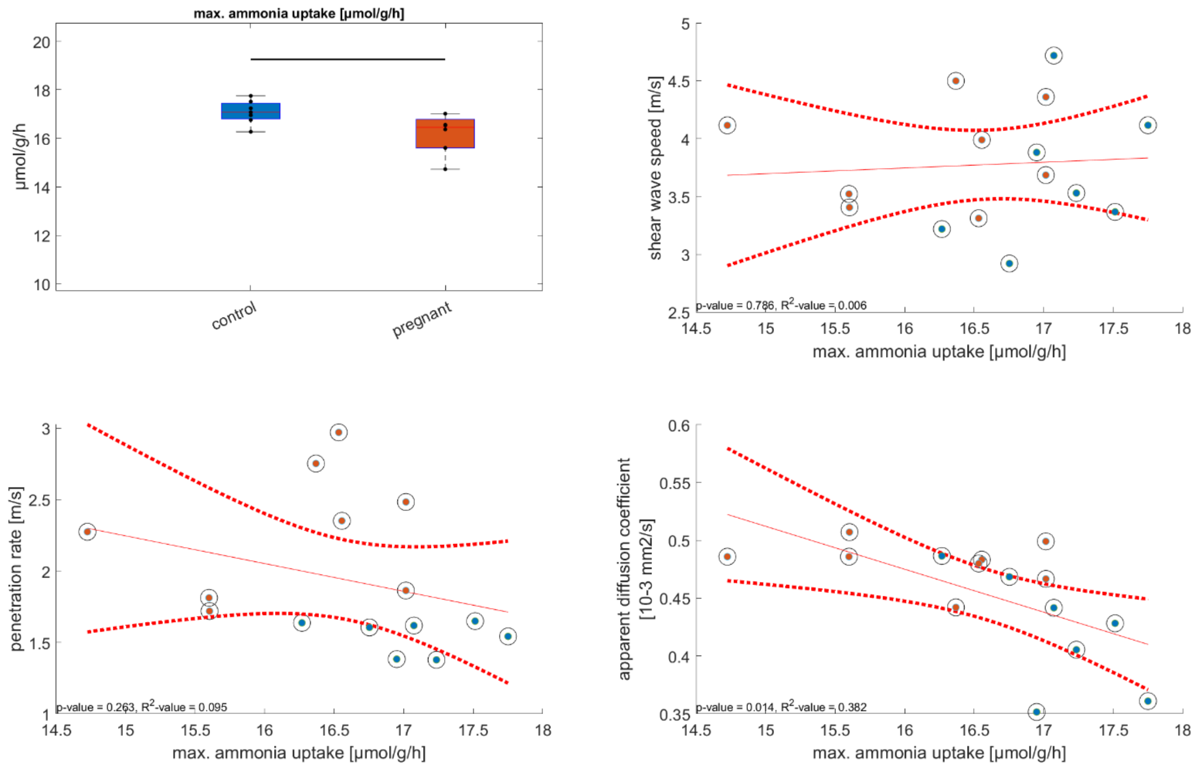

(E) Ketone body production

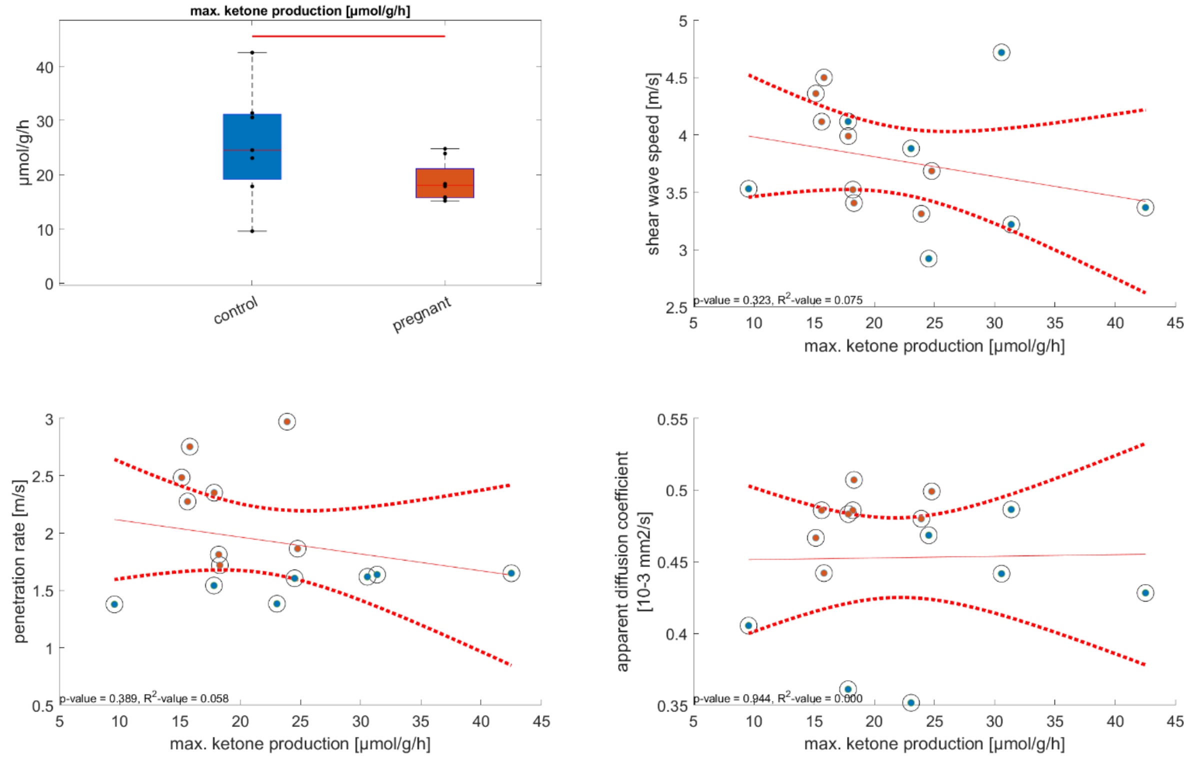

(F) Gluconeogenesis

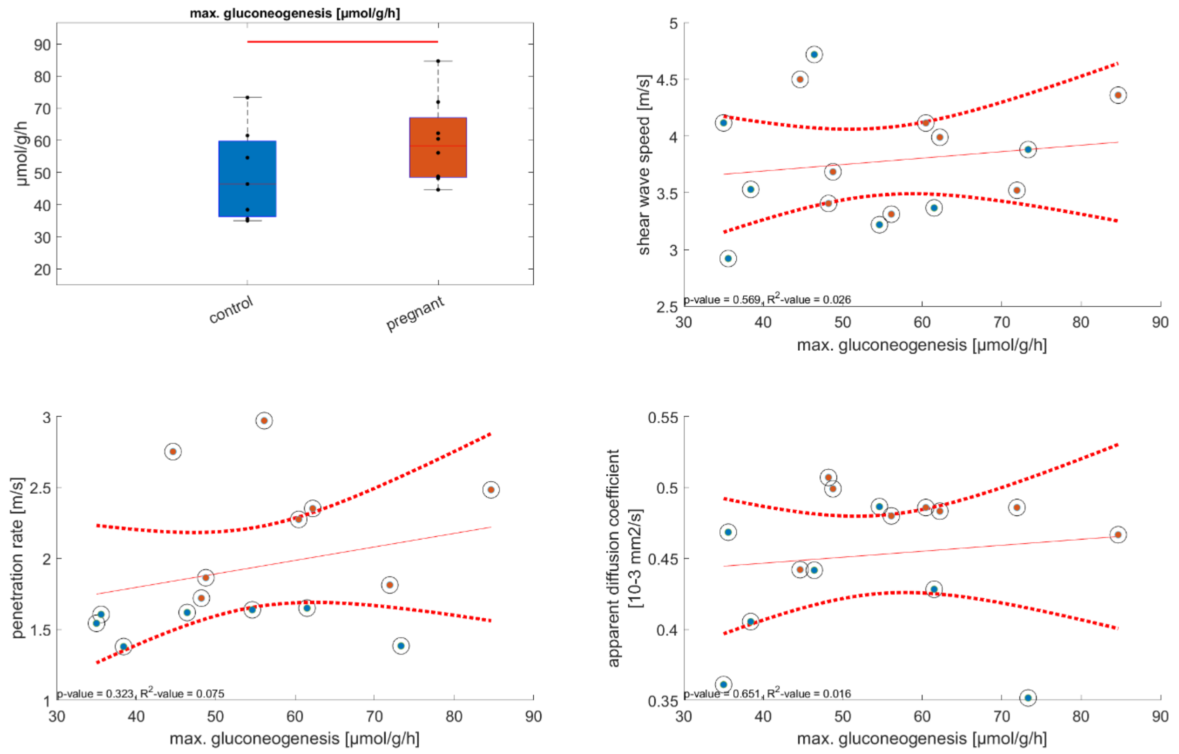

(G) Glucose uptake

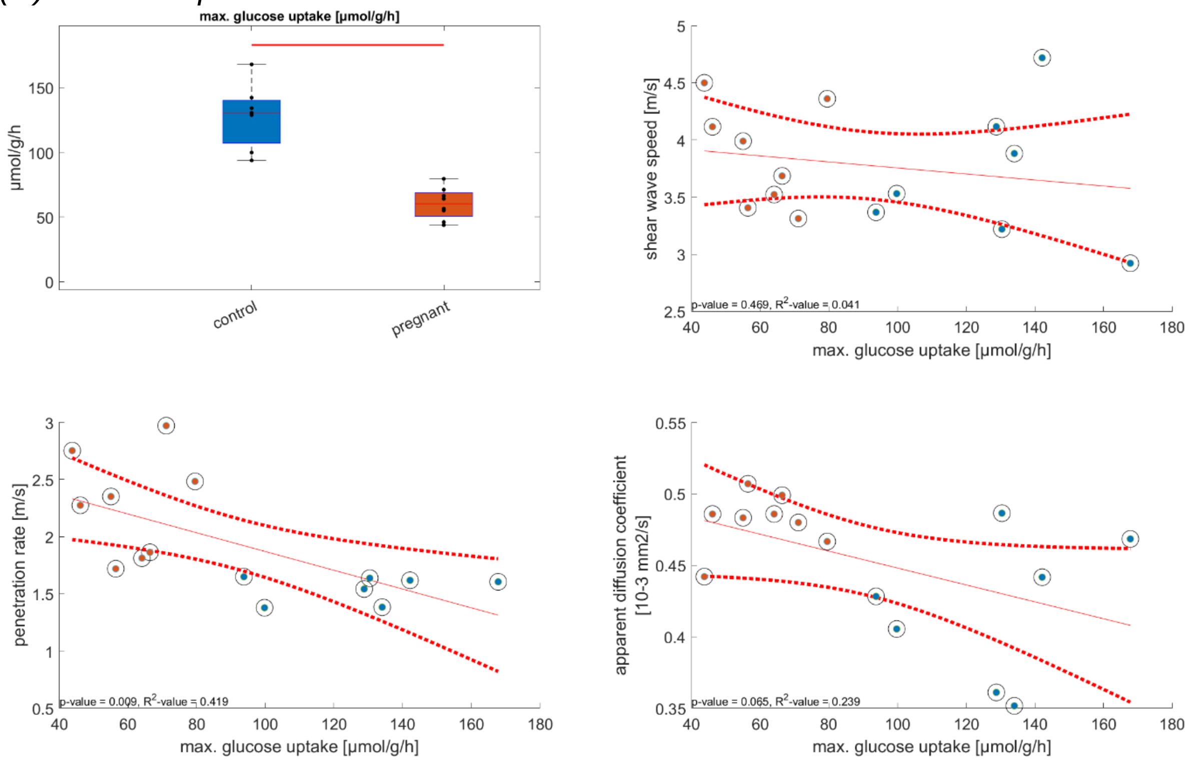

(H) Glutamate exchange

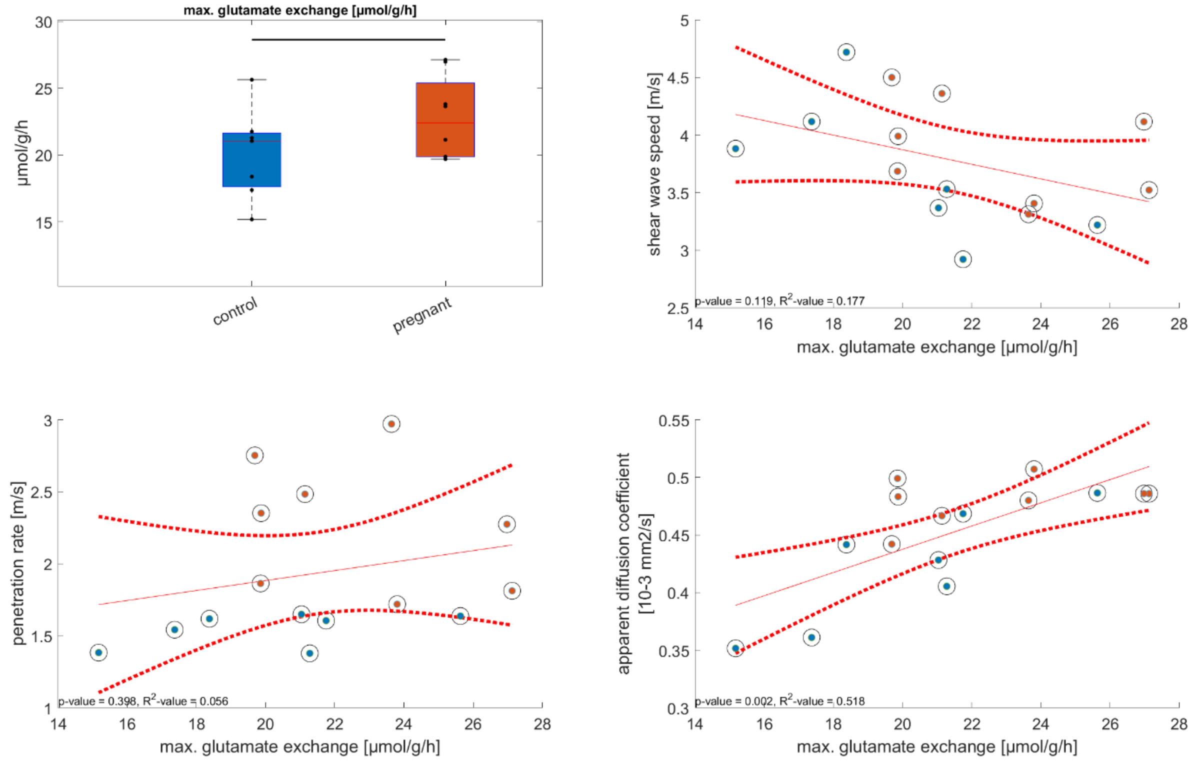

(I) Glutamine exchange

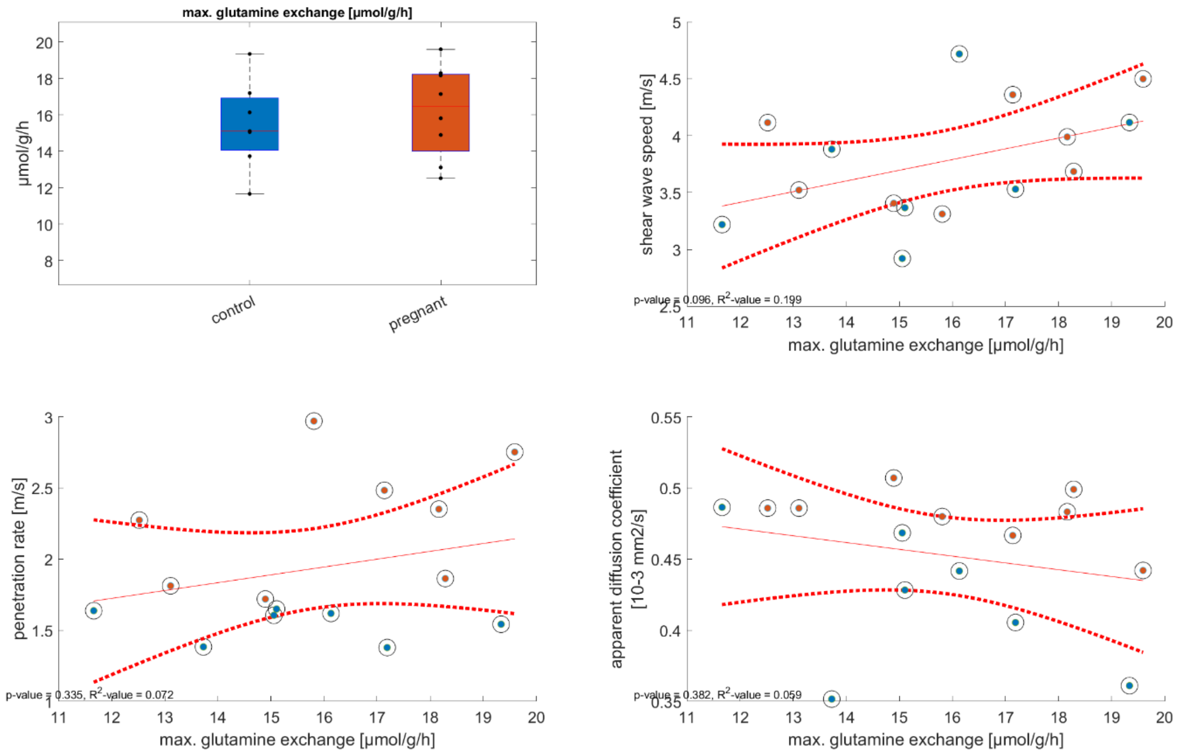

(J) Fatty acid uptake

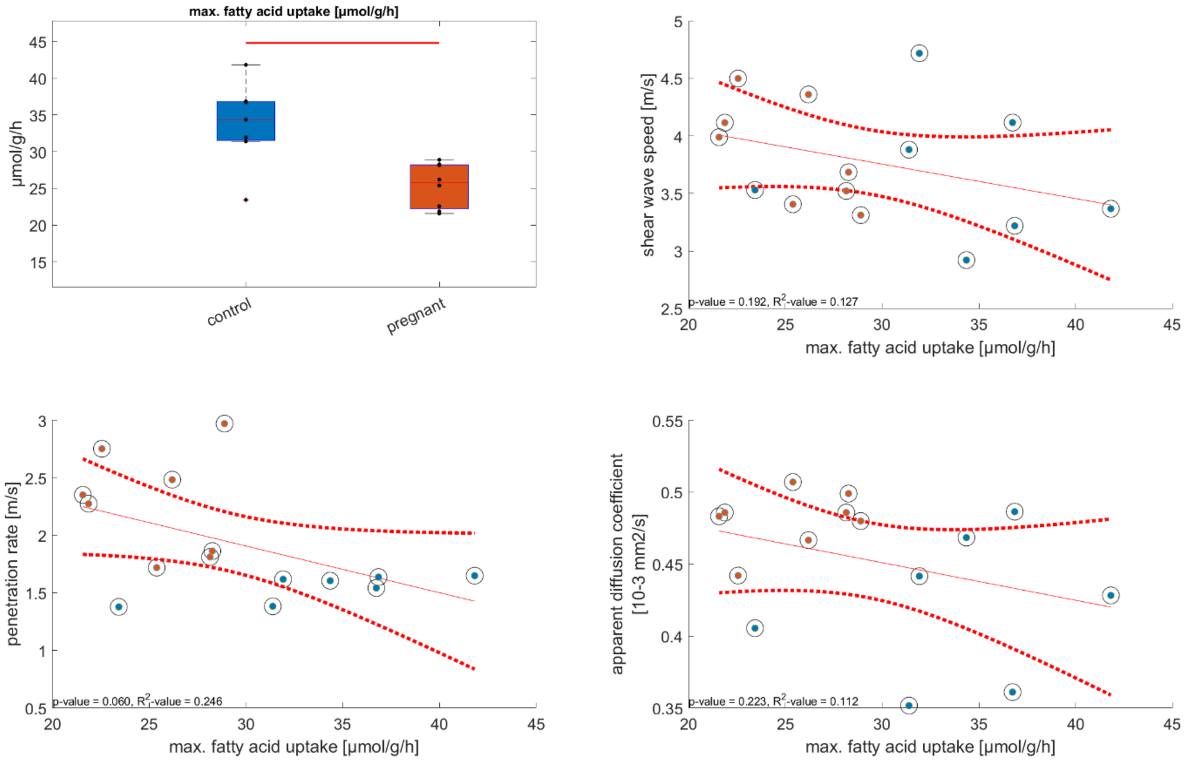

(K) Fatty acid synthesis

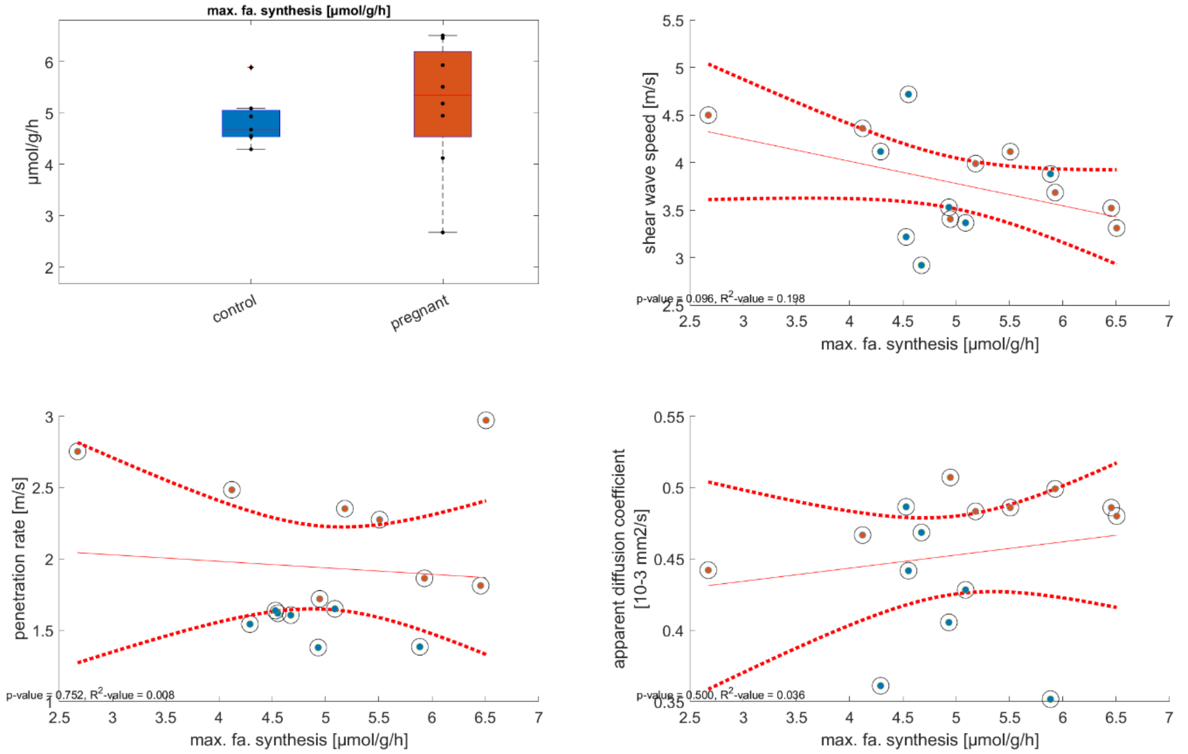

(L) Cholesterol synthesis

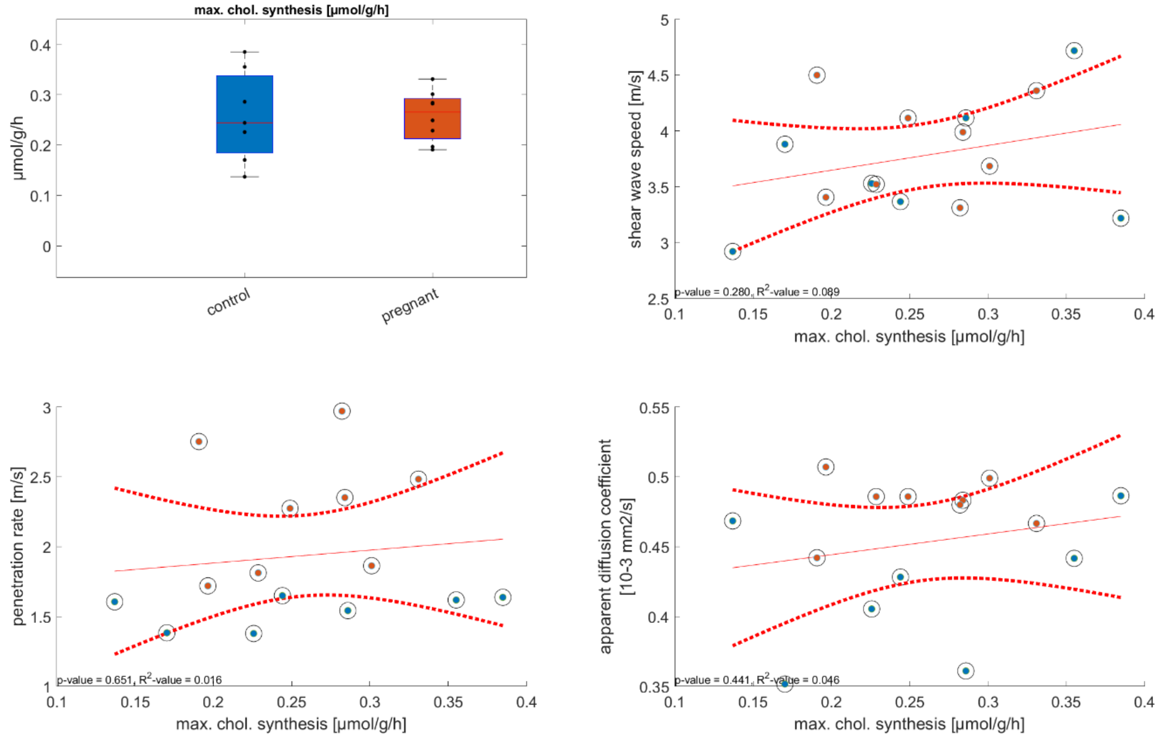

(M) Beta-oxidation

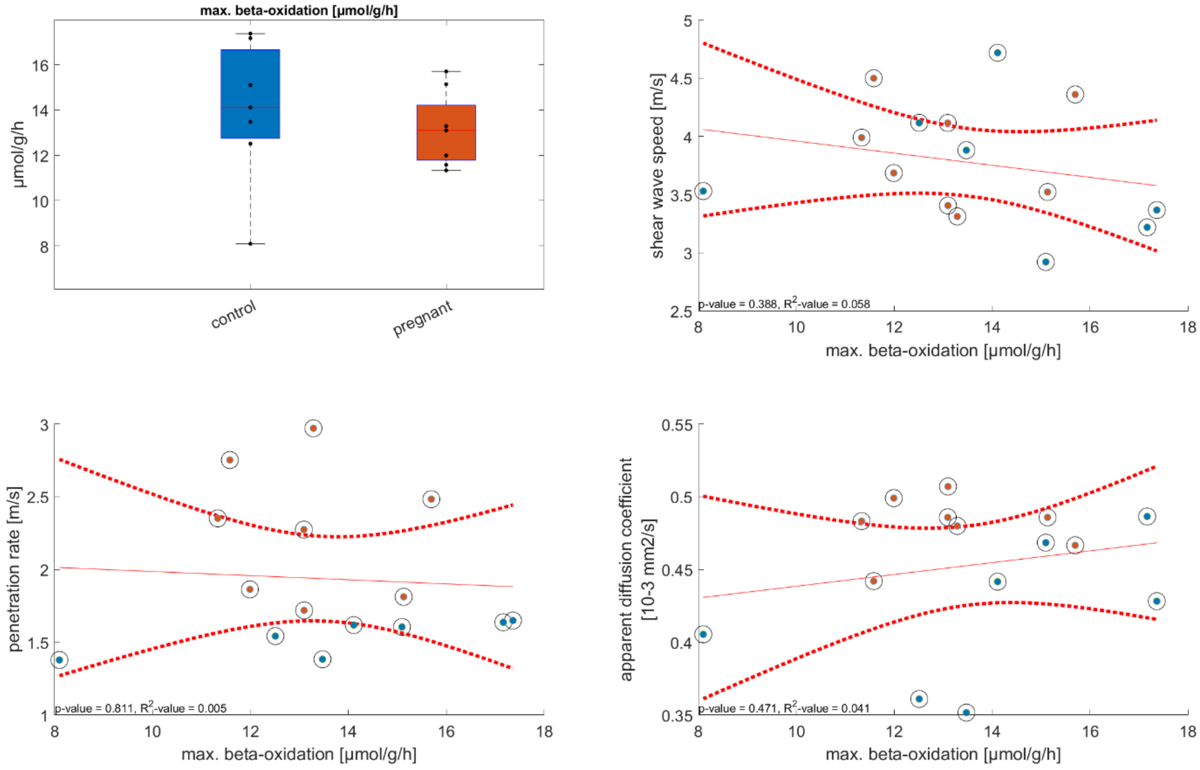

(N) Very-low-density lipoprotein (VLDL) production

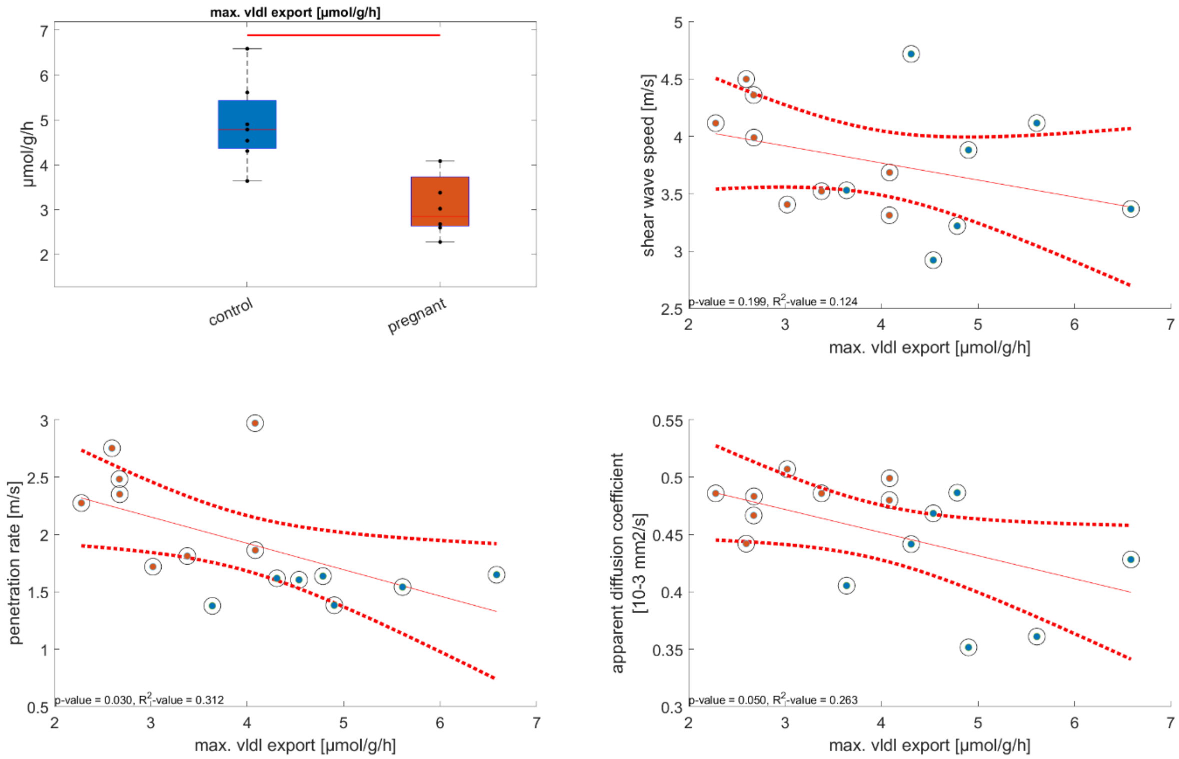

(O) Acetoacetate production

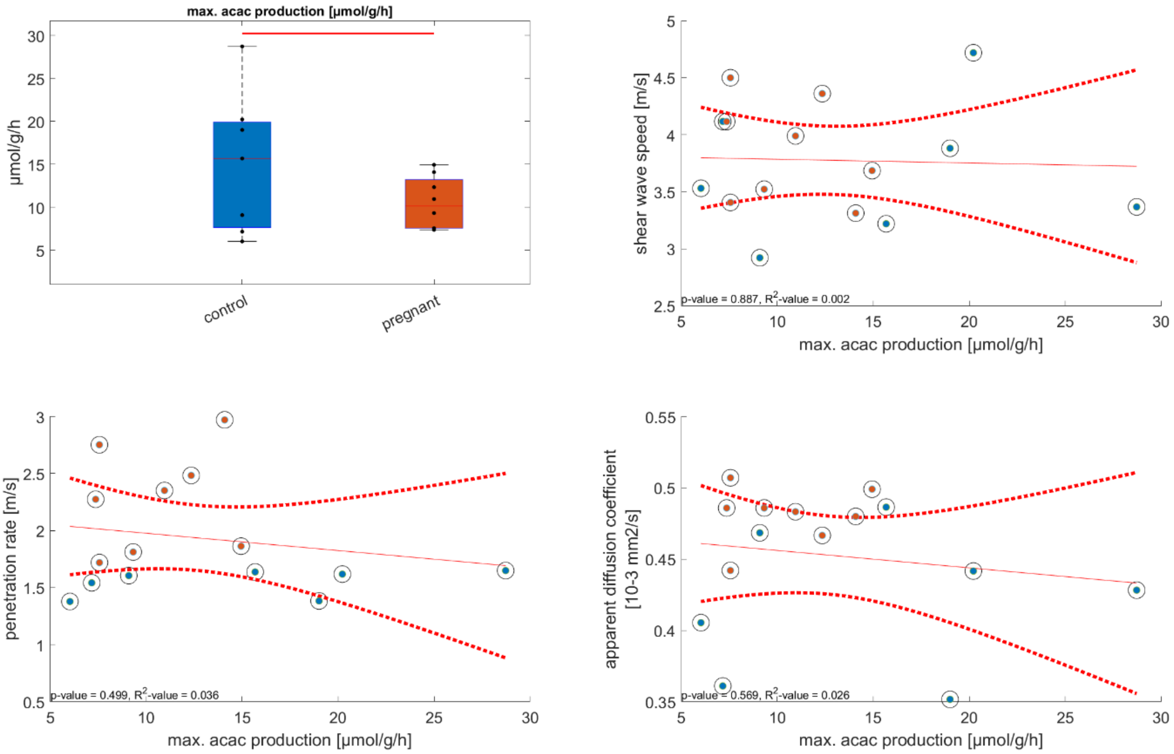

(P) Beta-hydroxybutyrate production

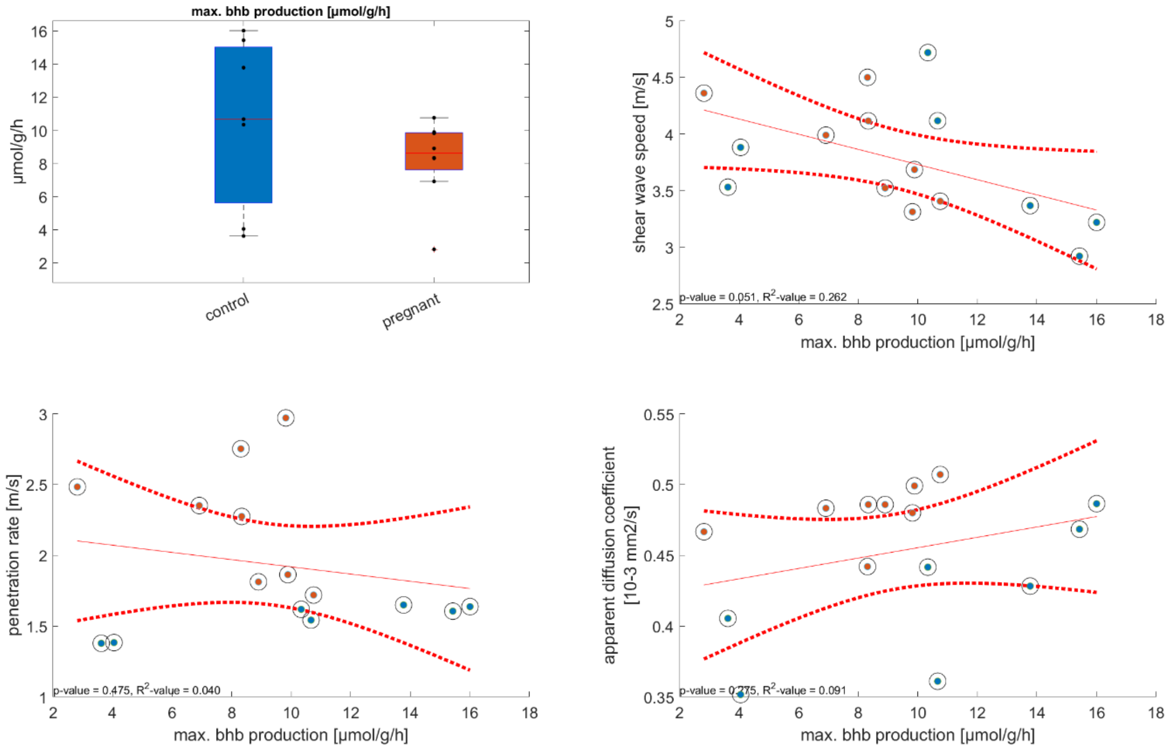

Supplementary Fig. S3 Correlations between the biophysical properties and the metabolic functions – Maximal capacities

The association between visco-elastic properties and the different metabolic functions was determined by a linear regression model. Linear regression fit and associated p-values for the association between visco-elastic properties (*c* in m/s, shear wave speed; *a* in m/s, penetration rate; and ADC in 10^{-3} mm^2/s, apparent diffusion coefficient) and the different metabolic functions are given below.

The differences in the metabolic functions between the control livers (n = 6) and the livers of pregnant animals (n = 6) are given by the box plots in the first panels. The center lines represent the median, the boxes represent the interquartile range, and the whiskers are defined by values within 1.5 times the interquartile range. Individual values for the different samples are represented as black dots. P-values are given above the brackets. Red and black brackets indicate significances with p-values <0.01 and <0.05, respectively. Orange and yellow brackets indicate a tendency with p-values <0.1 and <0.25, respectively. The 2nd to 4th panels show a linear regression model between the metabolic function and the visco-elastic properties. Red dotted lines indicate 95% confidence intervals of the linear model given by the solid red lines. Dots represent values of protein abundances and associated metabolic functions for individual samples (blue: control liver, orange: liver of pregnant rats). P- and R^2-values for linear regression are given in the lower right corner.

(A) Ethanol uptake

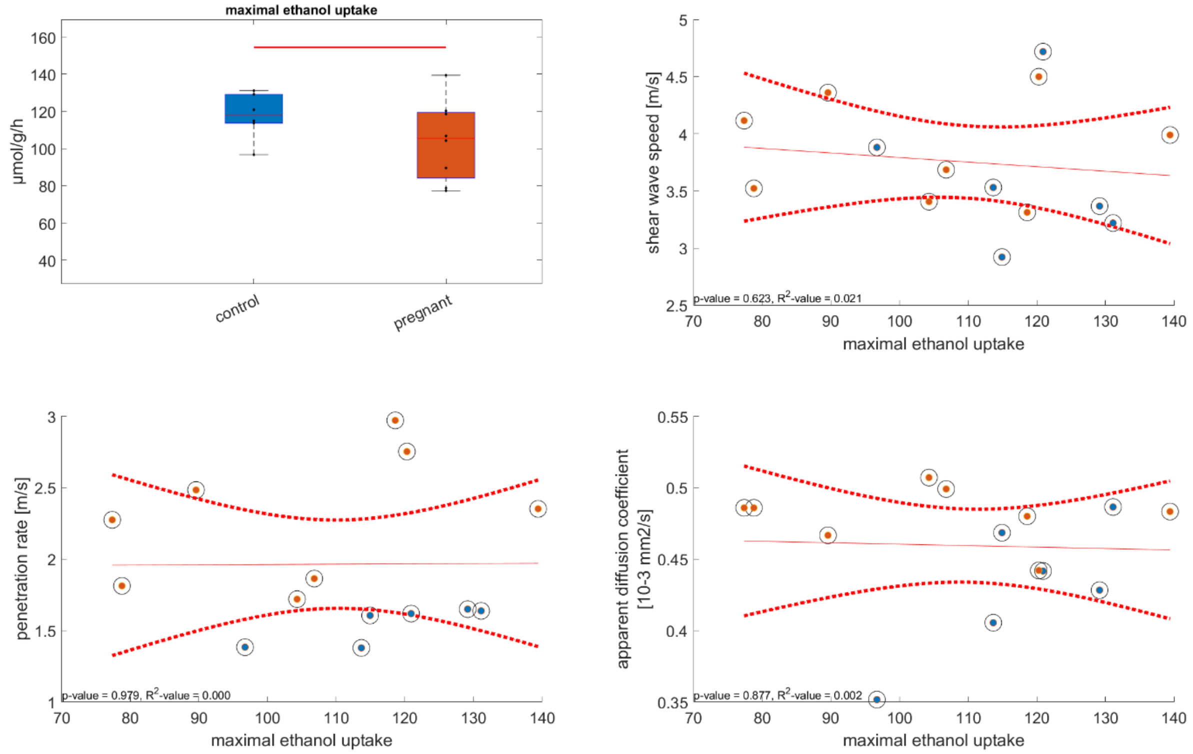

(B) Acetoacetate production

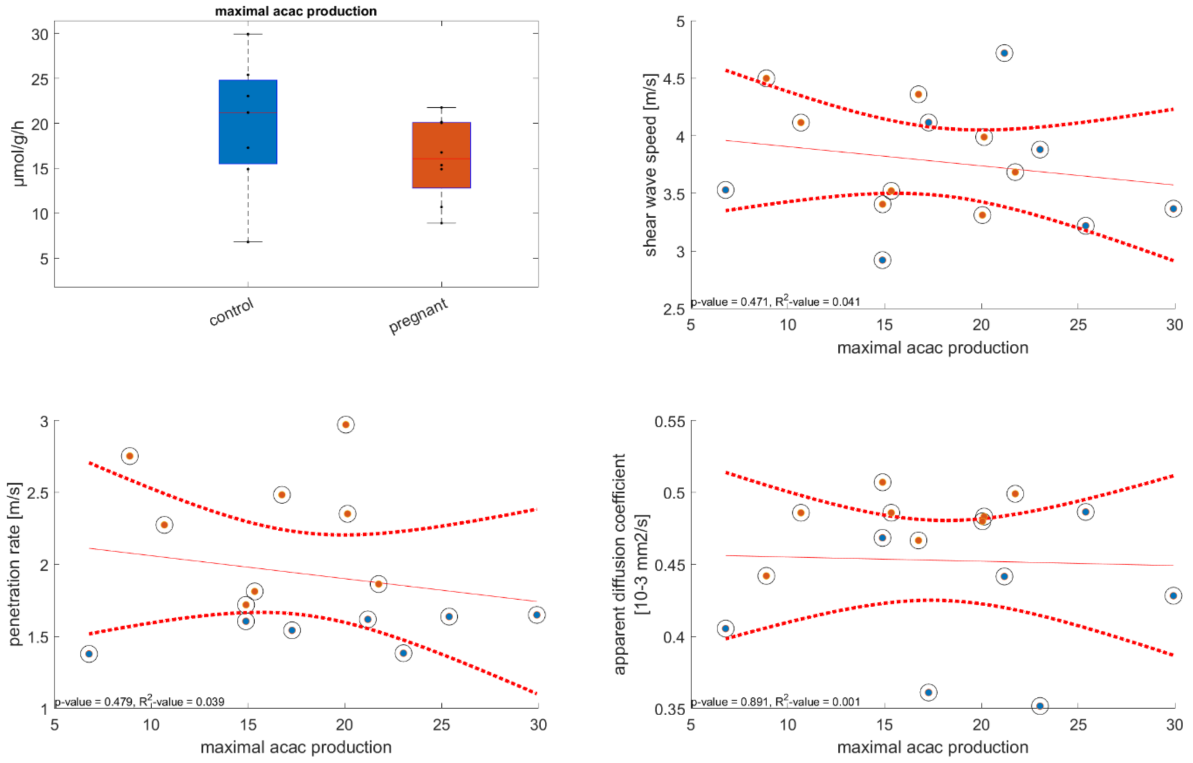

(C) Beta-hydroxybutyrate production

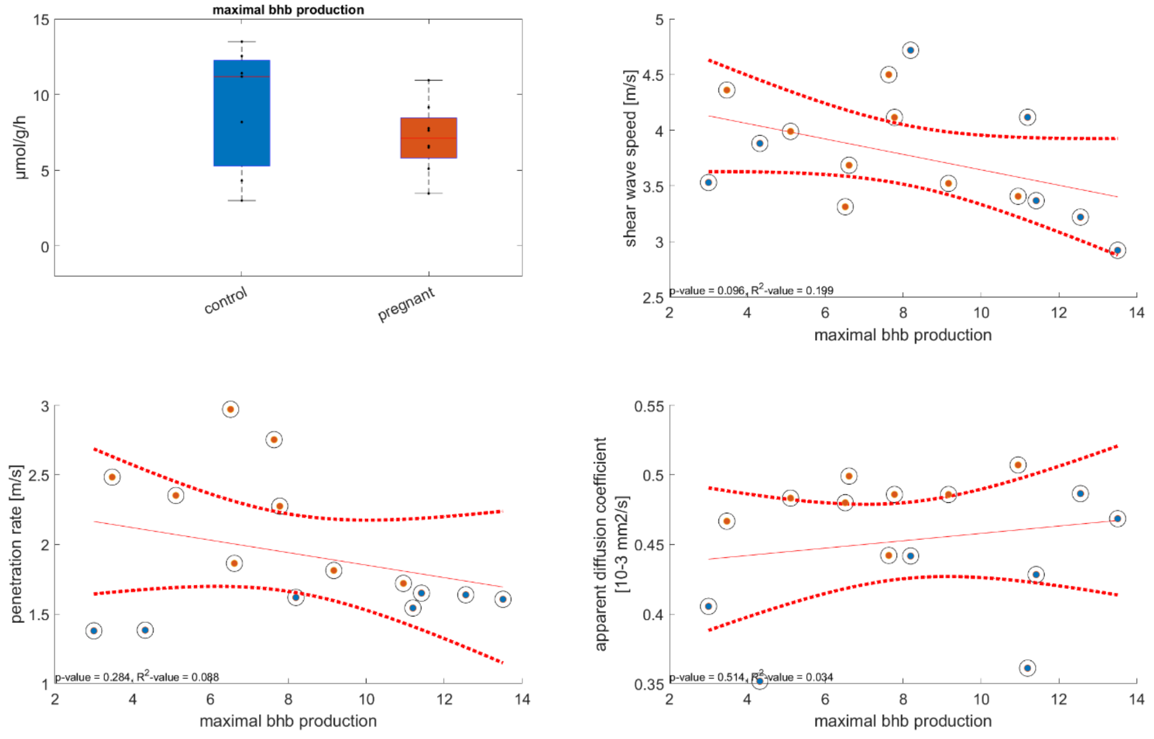

(D) Fatty acid uptake

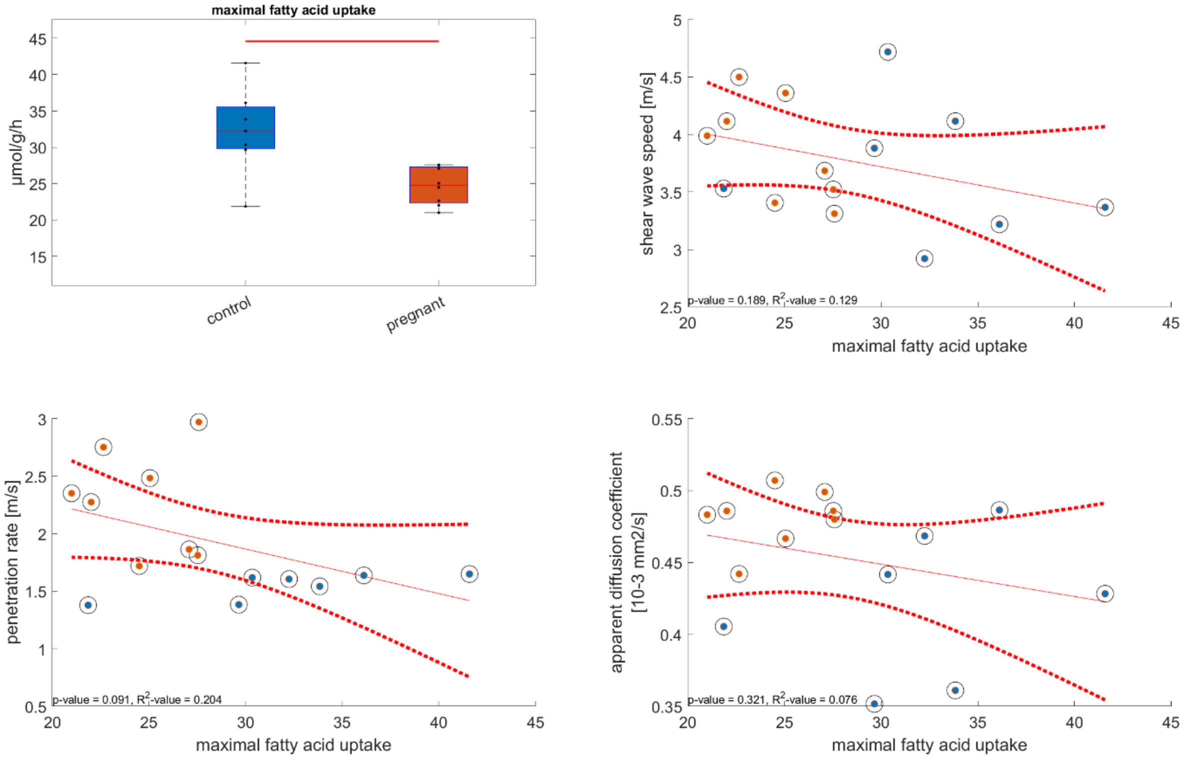

(E) Ketone body uptake

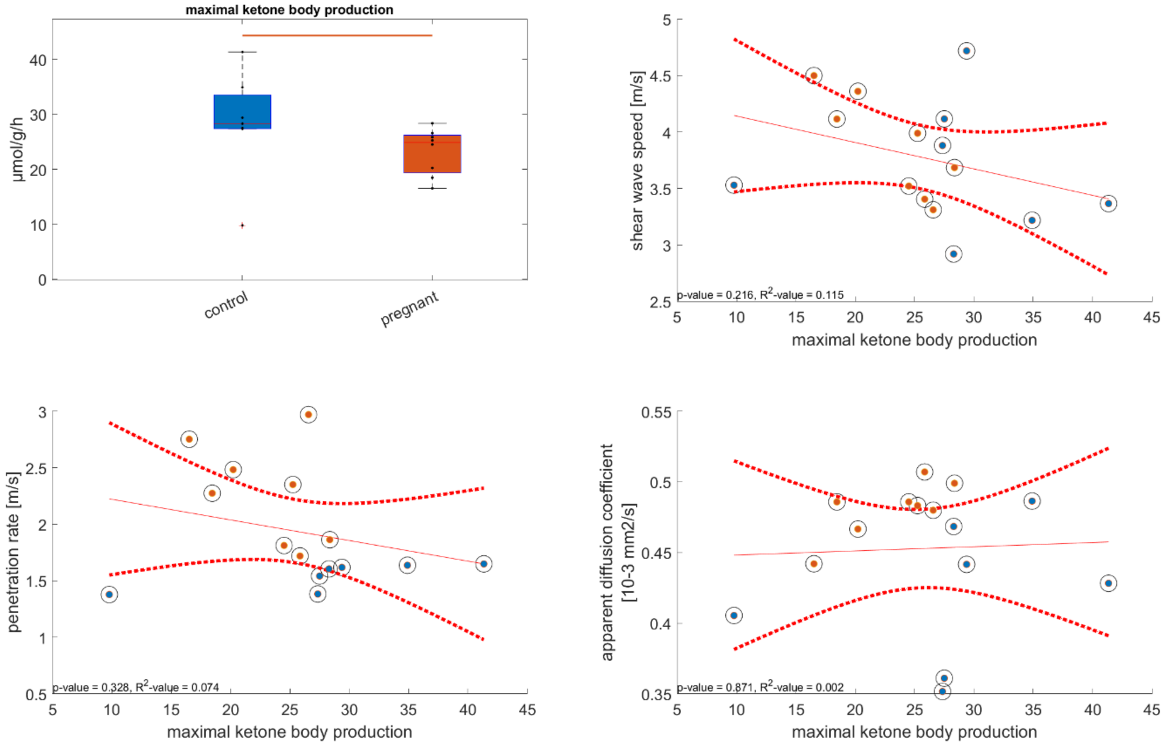

(F) TAG storage

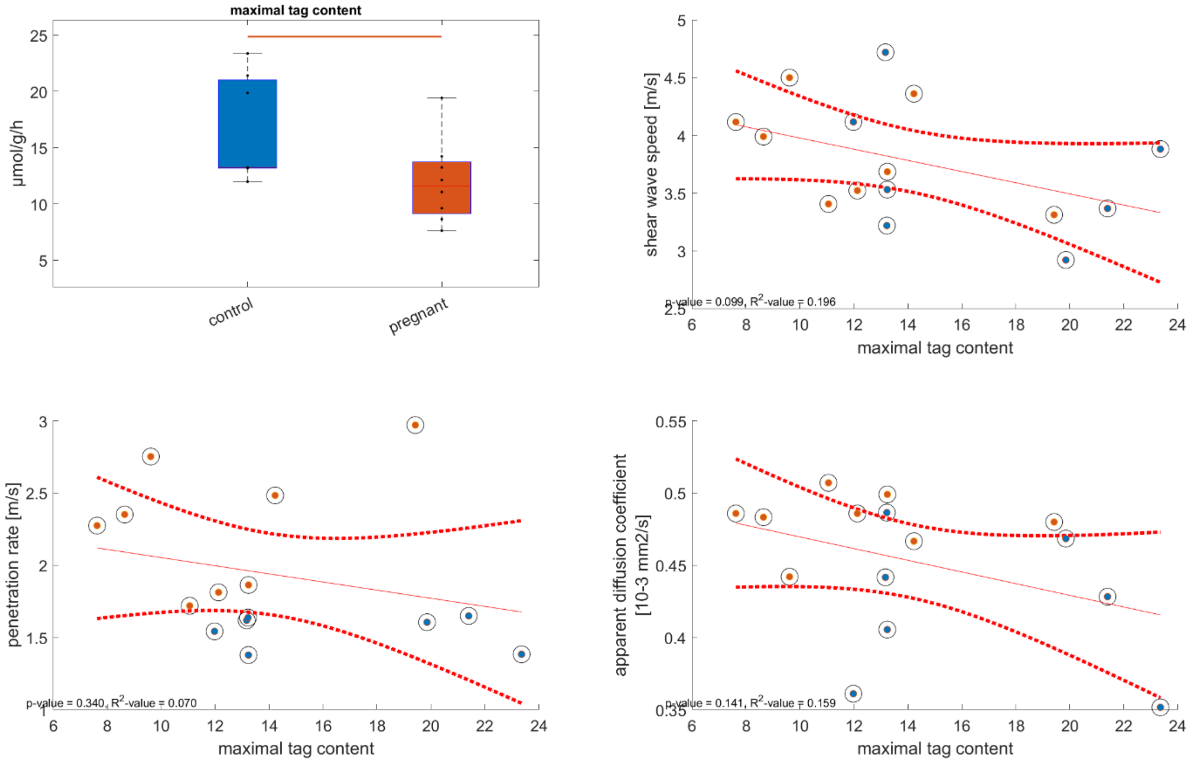

(G) TAG synthesis

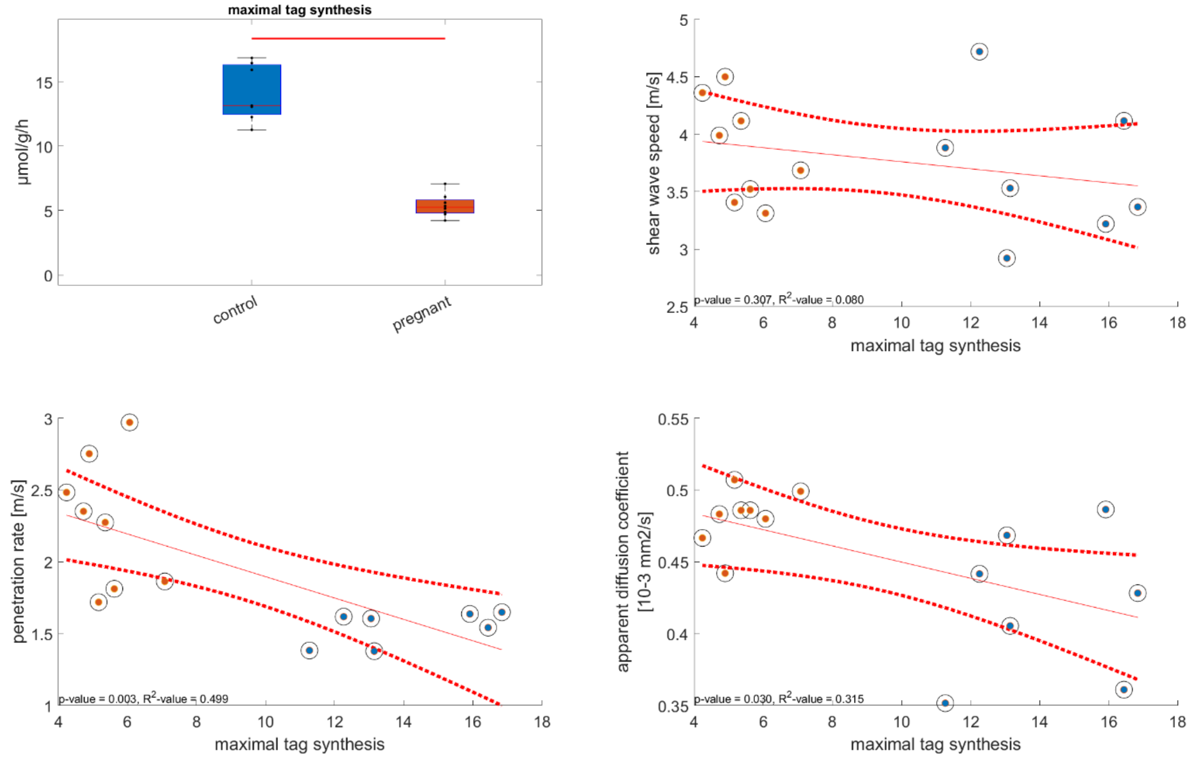

(H) VLDL production

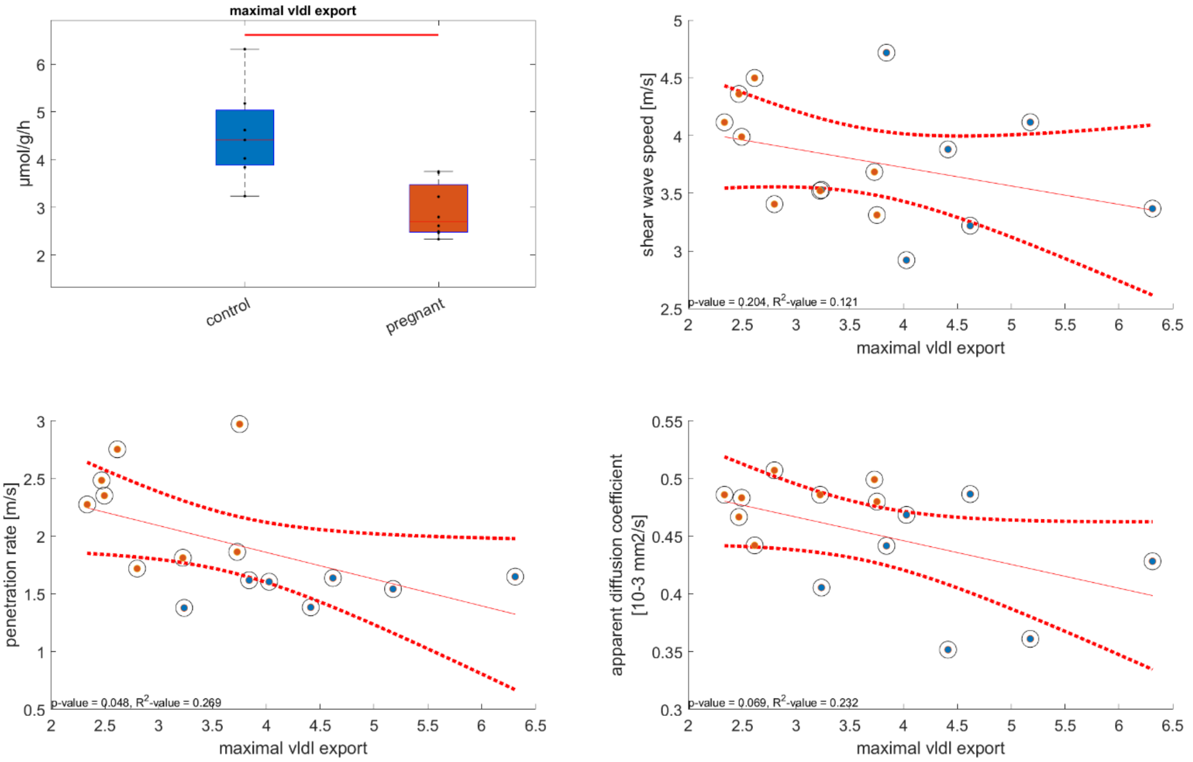

(I) Fructose uptake

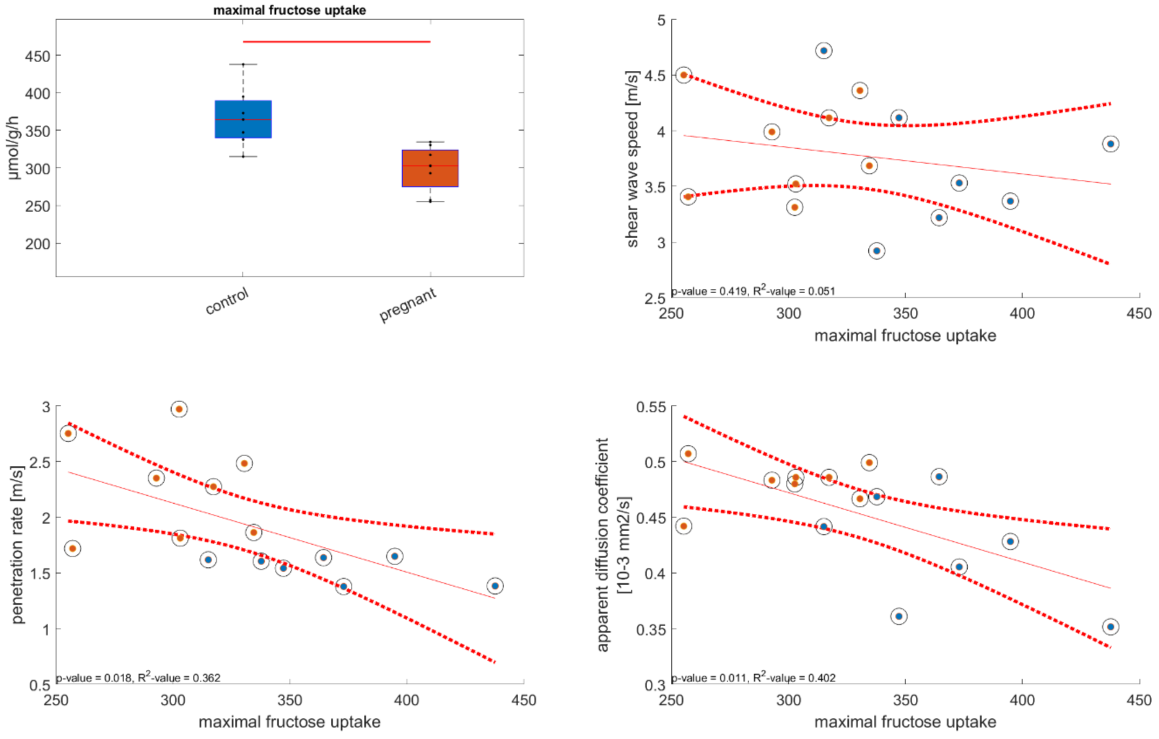

(J) Galactose uptake

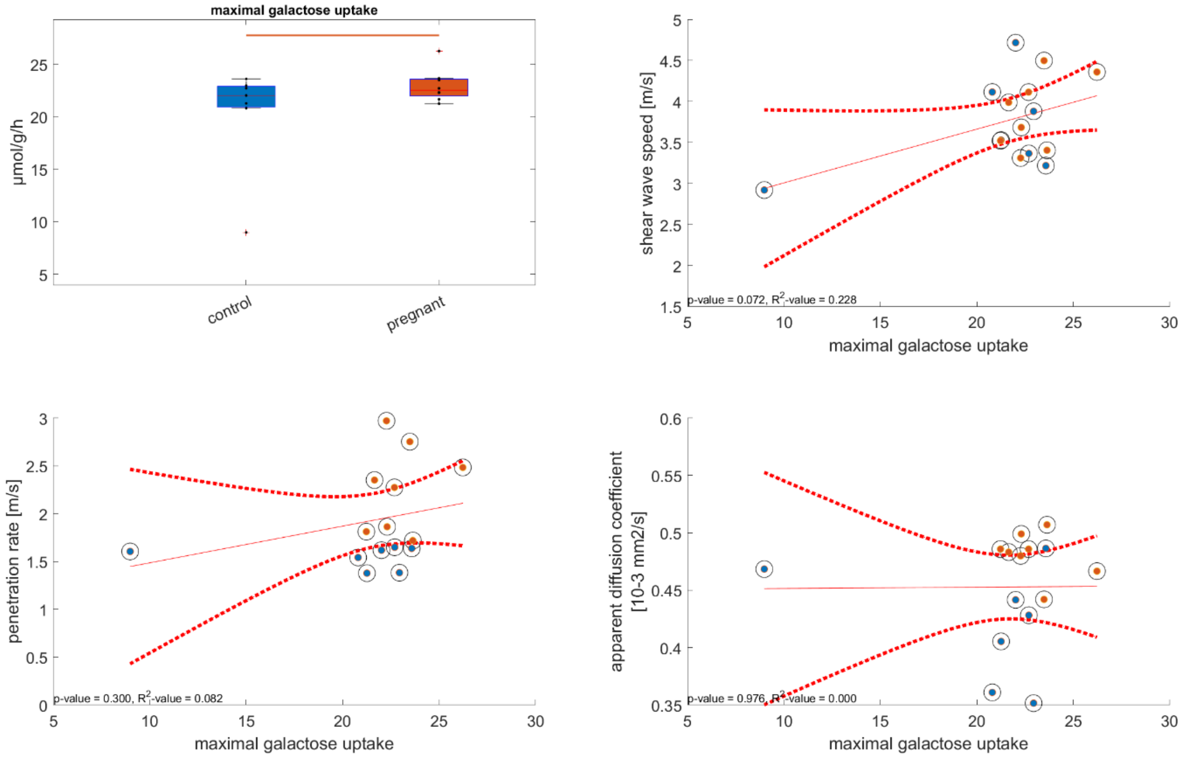

(K) Glycerol uptake

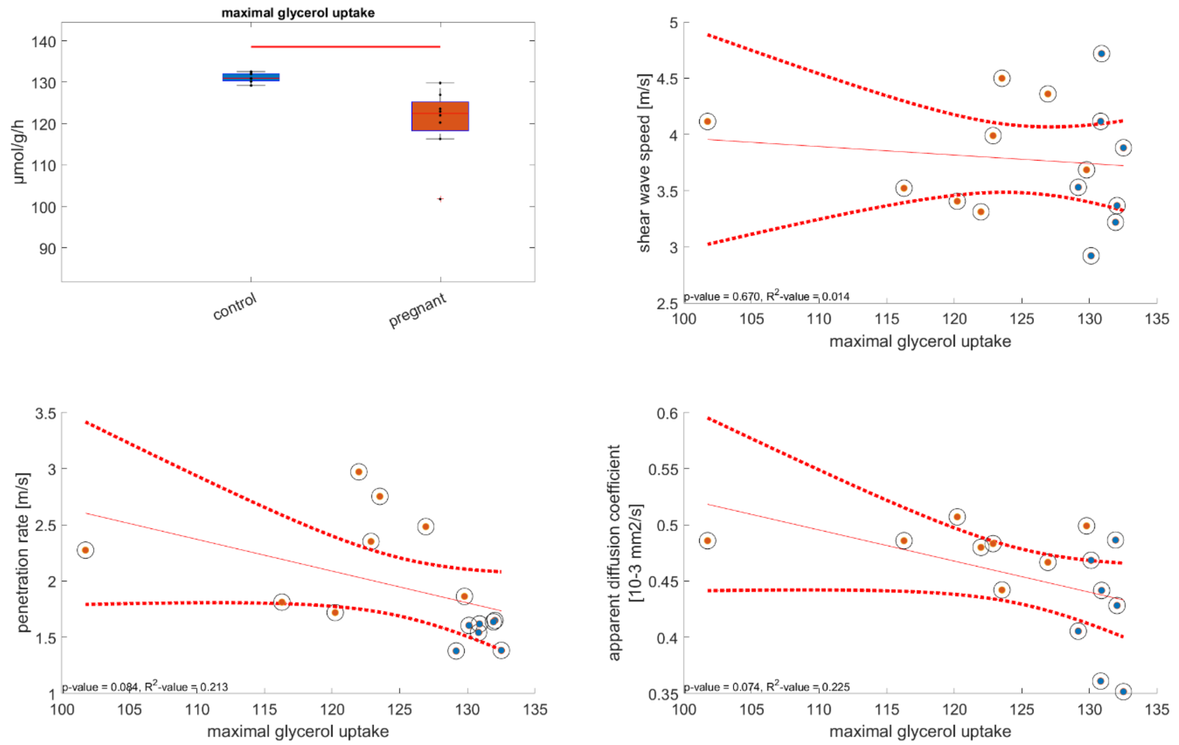

(L) Gluconeogenesis

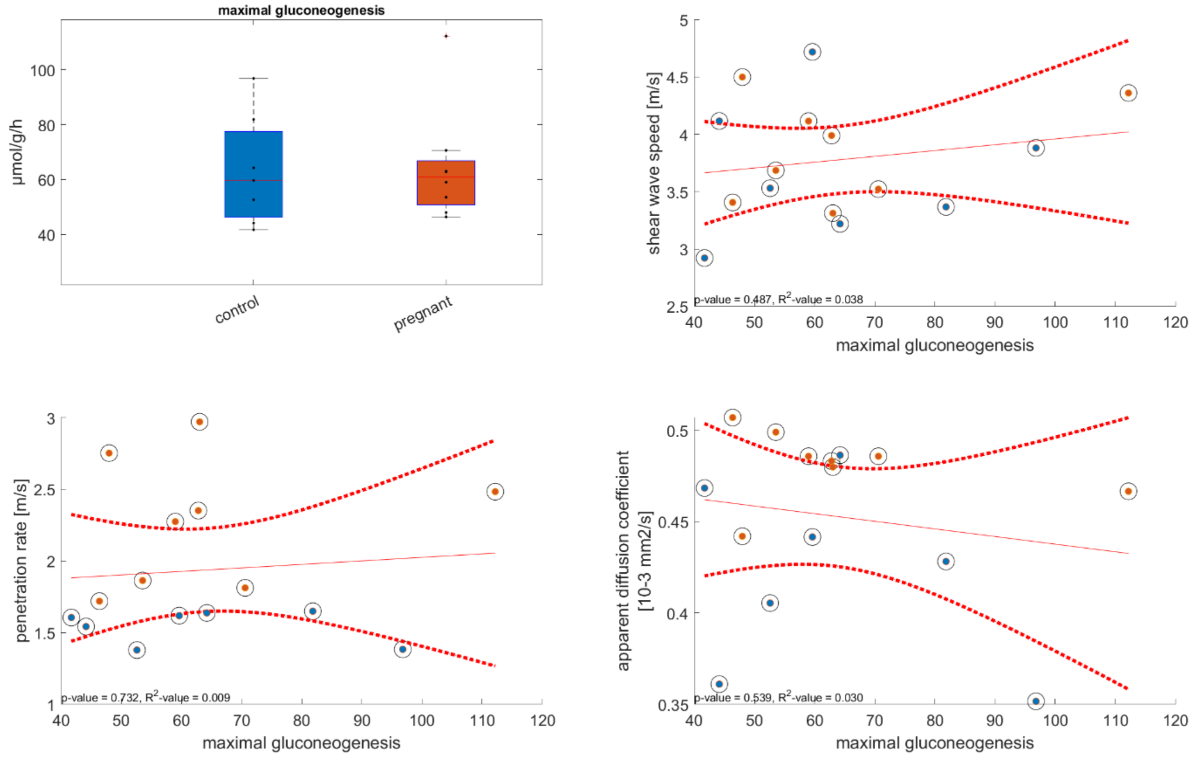

(M) Ammonia uptake

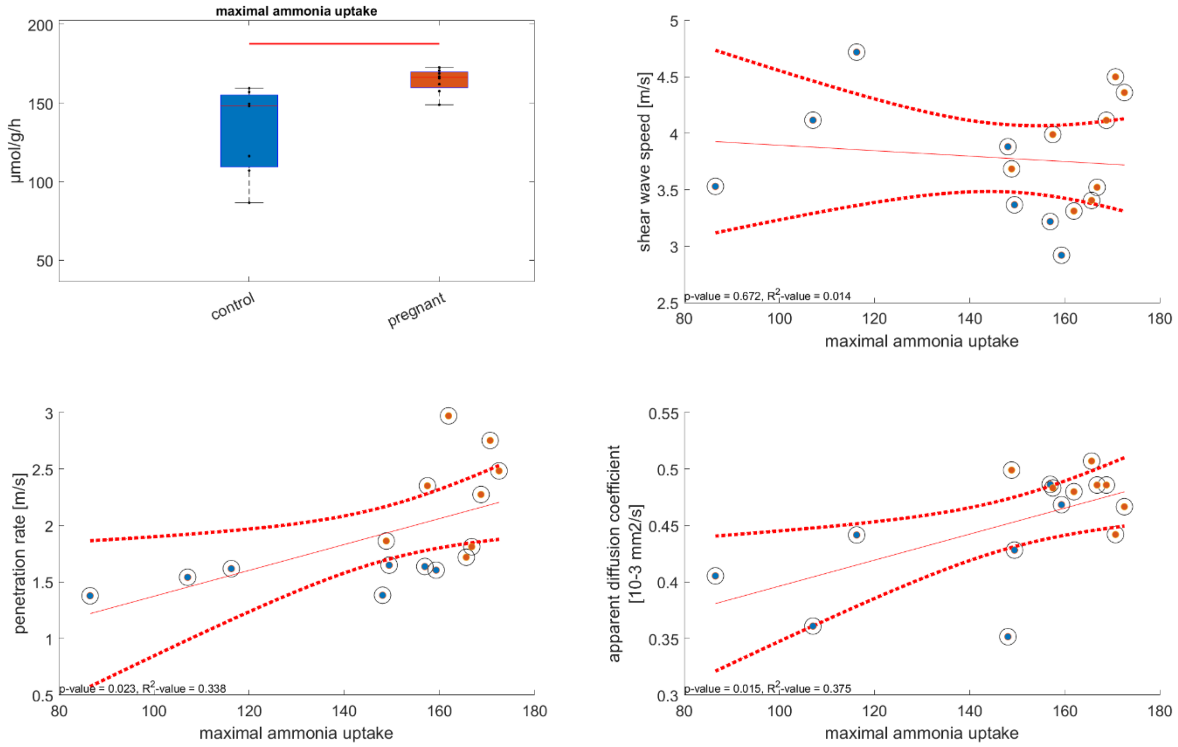

(N) Urea production

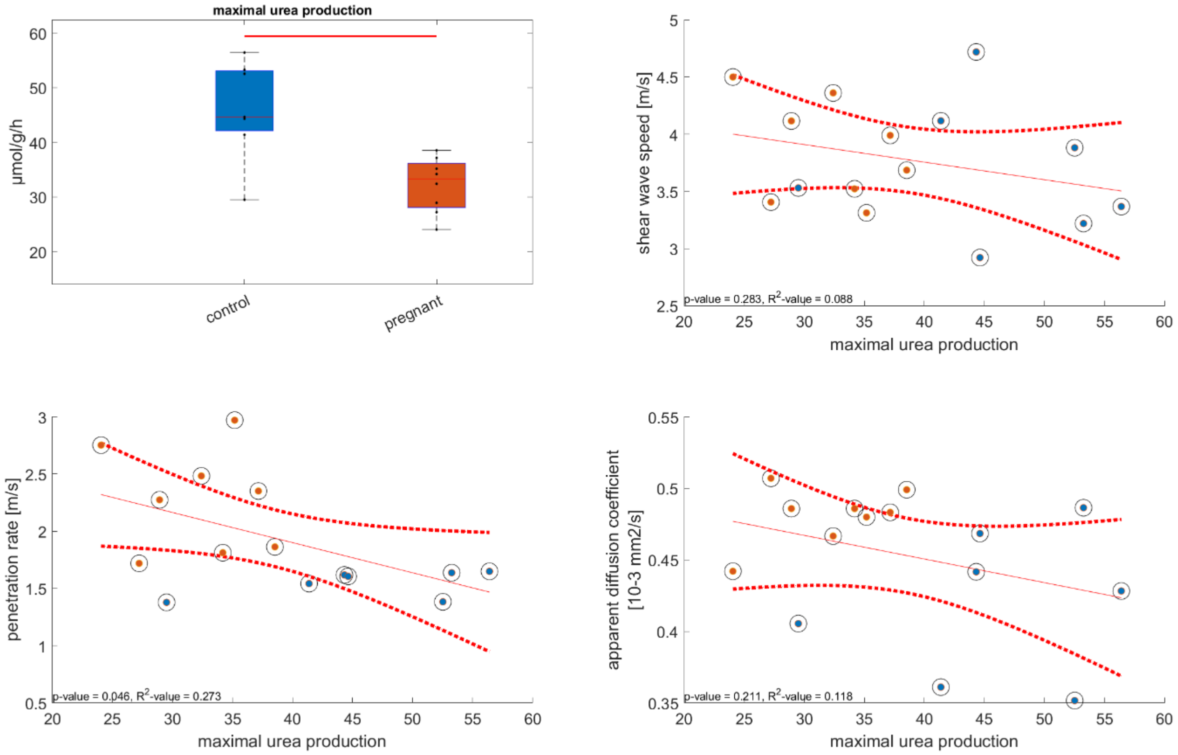

Supplement: Multimedia component 1 [file mmc1.pdf]
